# Absence of herb-drug interactions of mistletoe with the tamoxifen metabolite (E/Z)-endoxifen and cytochrome P450 3A4/5 and 2D6 in vitro

**DOI:** 10.1186/s12906-019-2439-2

**Published:** 2019-01-18

**Authors:** U. Weissenstein, M. Kunz, M. Oufir, J. T. Wang, M. Hamburger, K. Urech, U. Regueiro, S. Baumgartner

**Affiliations:** 1 Society for Cancer Research, Hiscia Institute, Arlesheim, Switzerland; 2 Iscador AG, Arlesheim, Switzerland; 3 Pharmaceutical Biology, Department of Pharmaceutical Sciences, University of Basel, Basel, Switzerland; 4 Institute of Integrative Medicine, Witten/Herdecke University, Herdecke, Germany

**Keywords:** Mistletoe (*Viscum album* L.), Iscador, Tamoxifen, Endoxifen, Hormonal therapy, Herb-drug interactions, Cytostasis, Cytotoxicity, Cell cycle, CYP3A4/5, CYP2D6

## Abstract

**Background:**

Women diagnosed with breast cancer frequently seek complementary and alternative (CAM) treatment options that can help to cope with their disease and the side effects of conventional cancer therapy. Especially in Europe, breast cancer patients use herbal products containing mistletoe (*Viscum album* L.). The oldest and one of the most prescribed conventional drugs for the treatment of estrogen receptor positive breast cancer is tamoxifen. Aside from positive clinical experience with the combination of tamoxifen and mistletoe, little is known about possible herb-drug interactions (HDIs) between the two products. In the present in vitro study, we investigated the effect of standardized commercial mistletoe preparations on the activity of endoxifen, the major active metabolite of tamoxifen.

**Methods:**

The estrogen receptor positive human breast carcinoma cell line MCF-7 was treated with (E/Z)-endoxifen hydrochloride in the presence and absence of a defined estradiol concentration. Each concentration of the drug was combined with fermented *Viscum album* L. extracts (VAE) at clinically relevant doses, and proliferation, apoptosis and cell cycle were analyzed. In parallel, possible inhibition of CYP3A4/5 and CYP2D6 was investigated using 50-donor mixed gender pooled human liver microsomes (HLMs).

**Results:**

VAE did not inhibit endoxifen induced cytostasis and cytotoxicity. At higher concentrations, VAE showed an additive inhibitory effect. VAE preparations did not cause inhibition of CYP3A4/5 and CYP2D6 catalyzed tamoxifen metabolism.

**Conclusions:**

The in vitro results suggest that mistletoe preparations can be used in combination with tamoxifen without the risk of HDIs.

**Electronic supplementary material:**

The online version of this article (10.1186/s12906-019-2439-2) contains supplementary material, which is available to authorized users.

## Background

Breast cancer accounts for nearly a quarter of all cancers in females and affects about 12% of all women during their lifetime [1]. Approximately 70–80% of all breast tumors are estrogen receptor (ER) positive [2], and hormonal therapy plays therefore an important role in the therapy of early stage and metastatic ER-positive breast cancer. The non-steroidal selective estrogen receptor modulator (SERM) tamoxifen is the oldest and most commonly used endocrine drug for the treatment of hormone-dependent breast cancer [3, 4].

Tamoxifen is a prodrug with a relatively low affinity for estrogen receptors. The compound is metabolized in the liver by cytochrome P450 isoforms CYP2D6 and CYP3A4/5 to active metabolites such as endoxifen (4-hydroxy-N-desmethyl-tamoxifen). Endoxifen competes with estrogen and binds to ERs with an almost 100-fold higher affinity than tamoxifen [5]. It shows pro- and anti-estrogenic activity by its binding to ERα and/or ERβ, which are ligand-activated intracellular transcription factors. The resulting nuclear complexes change the transcription of estrogen-responsive genes responsible for the generation of multiple growth-promoting signals [6].

Cancer patients, especially women with breast cancer, often use complementary and alternative therapies (CAM), as they conceive them as an important part of their coping with the disease [7, 8]. In Europe, pharmaceutical preparations derived from mistletoe (*Viscum album* L.) are widely used and registered as drugs in many countries [9]. Cancer patients use them frequently, mostly in addition to conventional therapies, with the aim to cope better with the side effects of conventional cancer treatment, to boost the immune system, to reduce symptoms, and to increase the quality of life. Clinical studies have reported on the benefits of *Viscum album* extracts (VAE) not only in breast cancer patients but also for other types of cancer [10–13].

As the prevalence of CAM use has increased, so have concerns about potential interactions with standard oncological drugs [14]. Therefore, it is important to provide information about the efficacy, safety and interactions of complementary mistletoe therapy. Earlier in vitro studies proved that mistletoe extracts had no negative effects on the cytostatic and cytotoxic activity of several common conventional chemotherapeutic drugs and the therapeutic antibody Herceptin (trastuzumab) when used at concentrations that are typical for clinical use [15, 16].

The present study aimed at investigating possible effects of clinically relevant doses of a standardized fermented *Viscum album* extract (VAE) on the in vitro activity of endoxifen in the ER-positive breast carcinoma cell line MCF-7. As the growth of MCF-7 cells is concentration-dependently influenced by estradiol, the experiments were carried out in the absence and presence of a defined estradiol concentration. In parallel, possible CYP3A4/5 and CYP2D6 inhibitions by VAE using pooled HLMs (human liver microsomes) from 50 donors and tamoxifen, dextromethorphan, and testosterone as substrates were investigated.

## Methods

### Chemicals and reagents

The aqueous, fermented mistletoe preparation Iscador M spec. 5 mg (VAEM, host tree *Malus domestica*, Lot 1404/4159/1, total mistletoe lectin concentration 309 ng/mL), mistletoe preparations Iscador P 10 mg (VAEP, host tree *Pinus sylvestris*, Lot 6187/02), Iscador M spec. 5 mg (VAEM, host tree *Malus domestica,* Lot 7054/0, total mistletoe lectin concentration 306 ng/mL), and Iscador Qu spec. 5 mg (VAEQu, host tree *Quercus robur/petraea,* Lot 7026/02, total mistletoe lectin concentration 444 ng/mL) were provided by Iscador AG (Arlesheim, Switzerland).

(E/Z)-endoxifen hydrochloride (4-hydroxy-N-desmethyltamoxifen hydrochloride), β-estradiol (E2) and dextrorphan-*d*_3_ were obtained from Sigma-Aldrich GmbH (Buchs, CH). Quinidine and testosterone were obtained from TCI Chemicals (Tokyo, Japan). Ketoconazole, (E/Z) endoxifen, tamoxifen, dextrorphan tartrate, 6-β-hydroxytestosterone were purchased from Cayman Chemical (Ann Arbor, MI, USA). Endoxifen-*d*_5_ was obtained from TLC standards (Vaughan, Canada). Dextromethorphan hydrobromide hydrate and testosterone-*d*_3_ were obtained from Biomol GmbH (Hamburg, Germany).

Pooled human liver microsomes (HLMs, 50 donors, mixed gender) were purchased from Bioreclamation IVT (Baltimore, USA). NADPH regenerating system containing solution A (26 mM NADP^+^, 66 mM glucose-6-phosphate, and 66 mM MgCl_2_ in H_2_O) and solution B (40 U/mL glucose-6-phosphate dehydrogenase in 5 mM sodium citrate) was supplied by Corning (Woburn, MA, USA). EDTA (99.9%), KH_2_PO_4_ (≥98.0%), K_2_HPO_4_ (≥99.0%), phosphoric acid were purchased from Sigma-Aldrich GmbH (Buchs, CH).

### Cell culture

The human breast carcinoma cell line MCF-7 was obtained from DSMZ (German Collection of Microorganisms and Cell Cultures, Braunschweig, Germany).

MCF-7 cells were kept growing in log phase in phenol red-free Eagle’s MEM (Sigma-Aldrich) supplemented with 10% FCS, 2 mM L-glutamine and 1% Penicillin-Streptomycin (Sigma-Aldrich) at 37 °C in an atmosphere of 5% CO_2_ and 100% humidity. For experiments, cells from subconfluent monolayers were trypsinized (Trypsin-EDTA, Sigma-Aldrich). All tests were performed on cells from which steroids were withdrawn as previously described [17], and with medium supplemented with 3% charcoal-stripped FCS (FCS/DCC, dextran-coated charcoal, Sigma-Aldrich), respectively. The maximal passage number after thawing was 21.

### Drug concentrations in the assays

The median endoxifen serum levels measurable in tamoxifen-treated early-stage breast cancer patients are between about 9 nM (poor metabolizers) and 67 nM (ultrarapid metabolizers) [18, 19].

Iscador concentrations representative for a subcutaneous application are 0.1 and 1 μg/mL. These concentrations are nearly equivalent to an injection of 5 mg Iscador when referring to the body weight or the circulating blood volume. Concentrations of about 10 μg/mL correspond to doses used for intravenous Iscador applications.

In preliminary experiments the optimal concentration range and incubation time regarding in vitro activity was determined for (E/Z)-endoxifen hydrochloride and VAEM. In the main experiments, (E/Z)-endoxifen hydrochloride was applied at concentrations between 0.1 nM (10^− 4^ μM) and 100 μM and combined with VAEM between 0.1 and 100 μg/mL, depending on the assay. For cytochrome P450 inhibition assays VAE concentrations up to 500 μg/mL were used.

### Proliferation assay

Steroid depleted cells (4 × 10^3^) were plated in triplicates in 96-well plates. After cell attachment for 4–6 h, (E/Z)-endoxifen hydrochloride was added at following concentrations: 0, 10^− 4^, 10^− 2^, 1, 100 μM. Each concentration of endoxifen was combined with VAEM at concentrations of 0, 0.1, 1, 10 and 100 μg/mL, respectively.

Cells were cultured for 7 days under standard culture conditions, and each experiment was performed in triplicate in the absence or presence of 0.5 μM estradiol.

The proliferation rate was measured 4 h after incubation with WST-1 reagent (Roche, Mannheim, Germany) using a Labsystems multiscan RC microplate reader. The upper limit of absorbance was 2.0–2.1. All values were expressed as percent inhibition of proliferation relative to cells cultured without estradiol (untreated control).

### Analysis of cell death

Steroid depleted cells (2 × 10^5^/well) were treated in 6-well plates for 5 days with 0, 0.1, 1.0 and 10 μM (E/Z)-endoxifen hydrochloride. Each endoxifen concentration was combined with VAEM at concentrations of 0, 0.1, 1, 10 and 100 μg/mL in the presence or absence of 0.5 μM β-estradiol.

To detect apoptotic cell death, 2 × 10^5^ cells were incubated with Annexin V-FITC and 7-AAD (BD Biosciences Pharmingen™, San Diego, CA, USA) at room temperature in the dark. Samples were analyzed in a FACS Calibur flow cytometer (BD Biosciences, San Jose, CA). Annexin V-FITC positive and 7-AAD negative cells were identified as early apoptotic, while late apoptotic/necrotic cells were Annexin V-FITC and 7-AAD double positive. Values were given in percent of total cell number.

### Cell cycle analysis

Steroid depleted cells (3 × 10^5^/well) were treated in 6-well plates for 3 days with 0 and 1 μM (E/Z)-endoxifen hydrochloride, each combined with VAEM at concentrations of 0, 10 and 100 μg/mL in the presence or absence of 0.5 μM β-estradiol.

The CycleTest™ Plus DNA Kit (BD Biosciences, San Jose, CA) was used according to manufacturer’s instructions for cell cycle analysis and DNA QC particles for quality control. Data were acquired with a FACS Calibur flow cytometer (BD Biosciences, San Jose, CA) and analyzed using the FlowJo 7.6.1 software (Ashland, OR, USA).

### Cytochrome 3A4/5 and 2D6 inhibition assays

Daily working solutions of substrates (tamoxifen, testosterone, dextromethorphan) were prepared in sample dilution buffer (5 μM EDTA in 100 mM potassium phosphate buffer PPB) from the DMSO stock solutions, with a final DMSO concentration of 0.1% (*v*/v) in the incubation mixture. The incubation mixture (total volume of 500 μL) was initially constituted of 100 mM PPB pH 7.4, 50-donor pooled HLMs (0.5 mg protein/ml), a NADPH regenerating system to initiate the reaction, and the potential inhibitors ketoconazole (0.1; 1; 2; 5; 10; 100 μM) as specific inhibitor of CYP3A4/5, quinidine (0.1; 1; 2; 5; 10; 100 μM) as specific inhibitor of CYP2D6, or test concentrations (0.1; 1; 5; 10; 100; 500 μg/mL) of the mistletoe preparations. The mixture was pre-incubated for 15 min at 37 °C with gentle stirring (300 rpm) using a BioShake iQ (Quantifoil Instruments GmbH, Jena, Germany). The reaction was then started by adding the corresponding substrates at 5 μM for each CYP450 (testosterone for CYP3A4/5, dextromethorphan for CYP2D6, and tamoxifen for both CYP3A4/5 and CYP2D6). Reactions were quenched after 30 min by adding 500 μL of ice-cold acetonitrile with the corresponding internal standard (IS), namely testosterone-d3 (200 ng/mL) for 6β-hydroxytestosterone, dextrorphan-d3 (200 ng/mL) for dextrorphan and endoxifen-d5 (200 ng/mL) for endoxifen). To detect possible chemical instability of compounds three negative control incubations were performed, namely without inhibitors, without inhibitors and microsomes, and without inhibitors, microsomes and NADPH. Positive control incubations were performed with specific inhibitors of each CYP. After incubation, samples were centrifuged (11,688 g, 4 °C) for 10 min, and 900 μL of supernatant were collected and dried under nitrogen flow (Evaporex EVX-96, Apricots Designs Inc., USA). Dried residues were redissolved in 200 μL of DMSO by shaking on a BioShake iQ (Quantifoil Instruments GmbH, Jena, Germany) at 1500 rpm for 30 min prior to UHPLC-MS/MS analysis of the corresponding metabolites, namely endoxifen for tamoxifen, 6-β-hydroxytestosterone for testosterone, and dextrorphan for dextromethorphan.

All CYP450 inhibition assays were analyzed using an UHPLC 1290 system coupled to a 6460 tandem mass spectrometer with an Agilent Jet Stream electrospray ionization source in positive mode (all Agilent Technologies, Waldbronn, Germany). All compounds were separated with a flow rate of 0.4 mL/min on a Phenomenex Kinetex XBC18 column (1.7 μm, 2.1 × 50 mm; Phenomenex, Torrance, CA, USA) heated at 55 °C and the mobile phases were: 10 mM ammonium formate with 0.05% formic acid as eluent A and acetonitrile with 0.05% formic acid as eluent B. Five μL injected were used for the multiple reaction mode (MRM) quantitation.

The source was operated with these optimized settings: nebulizer pressure at 30 psi, nozzle voltage at 0 V, sheath gas flow at 11 L/min, sheath gas temperature at 300 °C, drying gas flow at 10 L/min, drying gas temperature at 320 °C and capillary voltage at 3500 V. MRM transitions, fragmentor voltage and collision energy for each substrate, metabolite and corresponding IS are provided in supporting information Additional file 1: Table S1.

All CYP450 inhibition assays were performed in triplicate, and the results were expressed as % metabolite vs control (mean ± S.D.).

### Data analysis

For each combination of endoxifen and mistletoe extract, three to five independent experiments were performed. Data were analyzed with 2-way analysis of variance (ANOVA, Type 6 decomposition) using Statistica 6.0 (Statsoft Inc., Tulsa, USA). The protected Fisher LSD-test was used for pairwise comparisons. This procedure gives a good safeguard against false-positive as well as false-negative errors [20]. Limit of significance was defined as *p* ≤ 0.05.

All datasets generated or analyzed during this study were included as additional files in the supplementary information (Additional file 2: Data_proliferation_apoptosis_cell_cycle and Additional file 3: Data_CYP2D6_and_CYP3A4-5_inhibition assays).

## Results

### Proliferation

Given that estradiol has a mitogenic effect on hormone receptor positive tumor cells and therefore may interfere with the effects of the investigated drugs, we performed the experiments in both estrogen-depleted conditions and in the presence of a defined estradiol concentration.

**Fig. 1:**
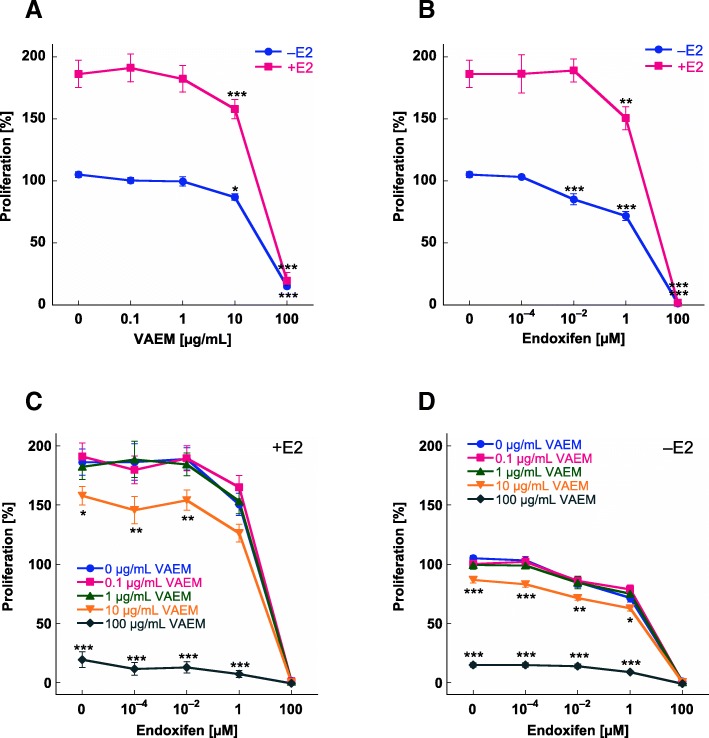
Concentration-dependent inhibition of MCF-7 cell proliferation by treatment for 7 days with (**a**) VAEM, (**b**) endoxifen, both in the presence or absence of 0.5 μM β-estradiol, (**c**) VAEM and endoxifen in the presence of 0.5 μM β-estradiol (E2) and (**d**) VAEM and endoxifen in the absence of β-estradiol. Cell growth kinetics was assessed with the WST-1 assay. Results are presented as % relative to untreated control (mean ± SE from three independent experiments). Significances are given relative to the VAEM untreated values (*p < 0.05, **p < 0.01, ***p < 0.001)

ER-positive breast cancer cells MCF-7 require estradiol for maximum growth in vitro. The proliferation rate of untreated cells grown in estradiol supplemented medium was about 90% higher compared to cells grown in steroid-depleted medium (Fig. 1).

VAEM, as well as endoxifen, showed a concentration-dependent inhibition of proliferation (Fig. 1a, b). In cells cultured in the presence or absence of estradiol, proliferation was significantly inhibited by VAEM concentrations ≥10 μg/mL (*p* < 0.05). 100 μg/mL VAEM reduced the proliferation of cells cultured in presence of estradiol to 20% and in cells cultured without estradiol to 15% (*p* < 0.001) (Fig. 1a). In cells grown without estradiol, proliferation was significantly inhibited by endoxifen concentrations ≥0.01 μM (*p* < 0.001). The anti-proliferative effect of 0.01 μM endoxifen was prevented by 0.5 μM estradiol. 1 μM Endoxifen significantly inhibited proliferation in cells cultured with or without estradiol (*p* < 0.01), and complete cell death was achieved at a concentration of 100 μM endoxifen under both conditions (Fig. 1b).

Mean proliferation values of MCF-7 cells after simultaneous application of VAEM and endoxifen with and without estradiol are shown in Fig. 1c, d. Concentrations of 0.1 and 1 μg/mL VAEM did not alter the cytostatic activity of endoxifen. At a concentration of 10 μg/mL VAEM distinctly inhibited the proliferation and significantly enhanced the anti-proliferative effect of 10^− 4^ and 10^− 2^ μM endoxifen in the presence (*p* < 0.01) (Fig. 1c) and of 10^− 4^ (*p* < 0.001), 10^− 2^ (*p* < 0.01) and 1 μM (*p* < 0.05) endoxifen in the absence of estradiol (Fig. 1d). The treatment with 100 μg/mL VAEM lowered the proliferation to less than 10–20% under both conditions.

### Apoptosis

**Fig. 2:**
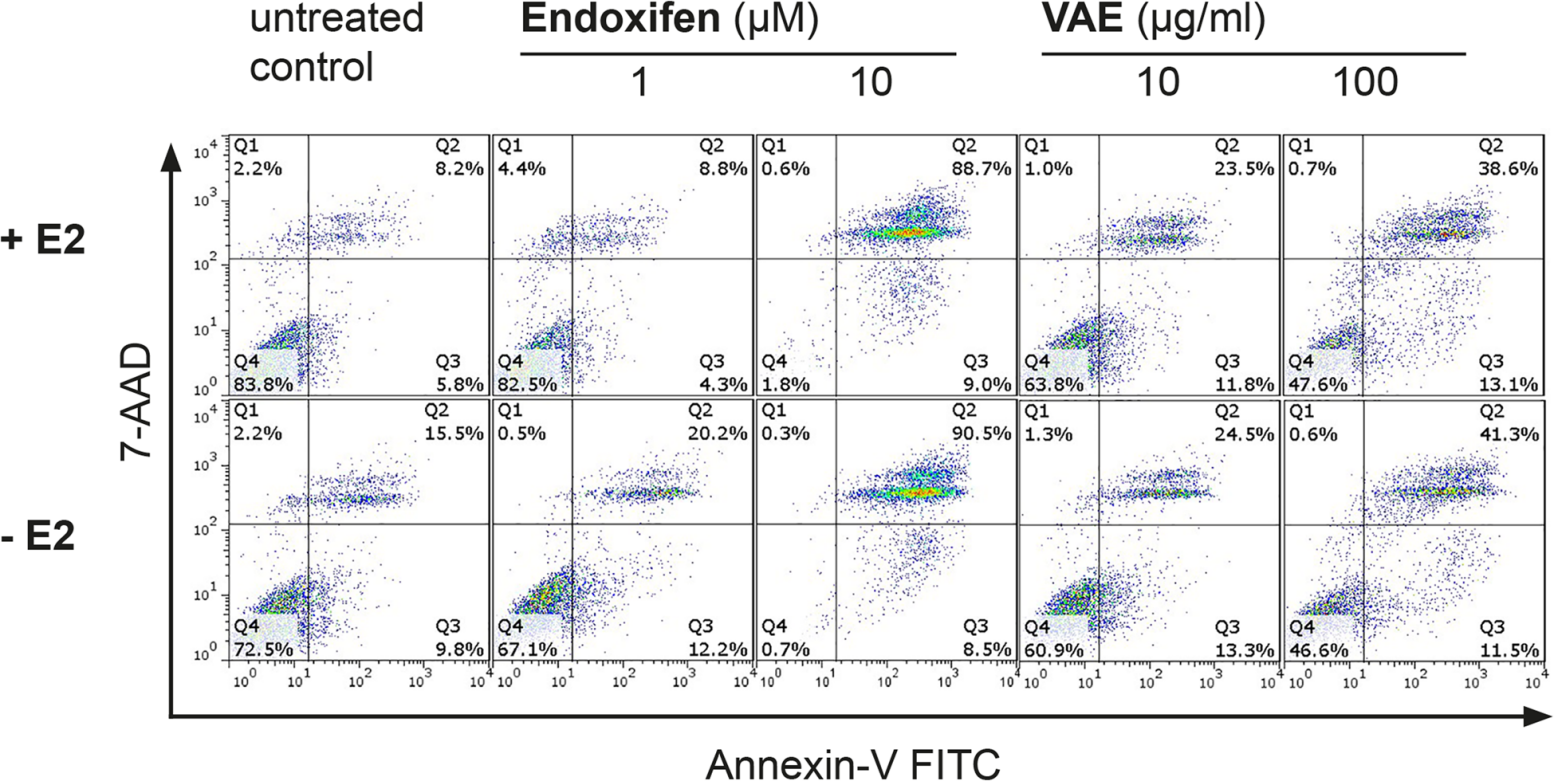
Apoptosis induction in MCF-7 cells by treatment with endoxifen or VAEM for 5 days. Flow cytometric analysis of apoptotic death in MCF-7 cells labeled with annexin-V FITC and 7-AAD in the presence (top) and absence (bottom) of 0.5 μM β-estradiol (E2). This figure is representative of three independent experiments. The percentages in the graphs represent the percentage of cell numbers in each quadrant. Q1 and Q2: late apoptotic/necrotic cells, Q3: early apoptotic cells, Q4: viable cells

Annexin V/7-AAD dual staining was used to evaluate the type of cell death induced by treatment with VAEM, endoxifen and their combinations in MCF-7 cells cultured in the presence or absence of estradiol (Fig. 2).

**Fig. 3:**
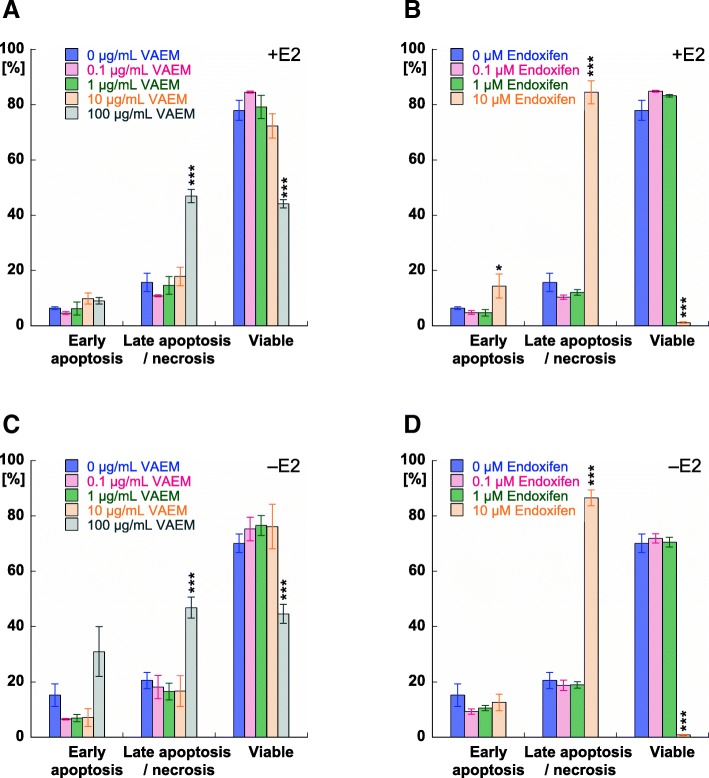
Apoptosis induction (%) in MCF-7 cells by endoxifen and VAEM, respectively. Mean values (±SE) of early apoptosis, late apoptosis/necrosis and viable cells after 5 days treatment with (**a**) VAEM in the presence of 0.5 μM β-estradiol (E2), (**b**) endoxifen in the presence of 0.5 μM β-estradiol, (**c**) VAEM in the absence of 0.5 μM β-estradiol and (**d**) endoxifen in the absence of 0.5 μM β-estradiol are presented. Significance values are given relative to the untreated control (*p < 0.05, **p < 0.01, ***p < 0.001)

VAEM concentrations between 0.1 and 10 μg/mL had no effect on the viability of MCF-7 cells cultured with or without estradiol. Under both treatment conditions, VAEM at 100 μg/mL significantly reduced the number of viable and increased the amount of late apoptotic/necrotic cells (*p* < 0.001) (Fig. 3a, c).

Endoxifen at a concentration of 10 μM was highly cytotoxic. A small but significant increase in early apoptosis (*p* < 0.05) in the presence of estradiol, and a massive increase of the amount of late apoptotic/necrotic cells was detected after treatment with 10 μM endoxifen in cells cultured in the presence or absence of estradiol (*p* < 0.001). Lower endoxifen concentrations did not significantly affect cell viability (Fig. 3b, d).

**Fig. 4:**
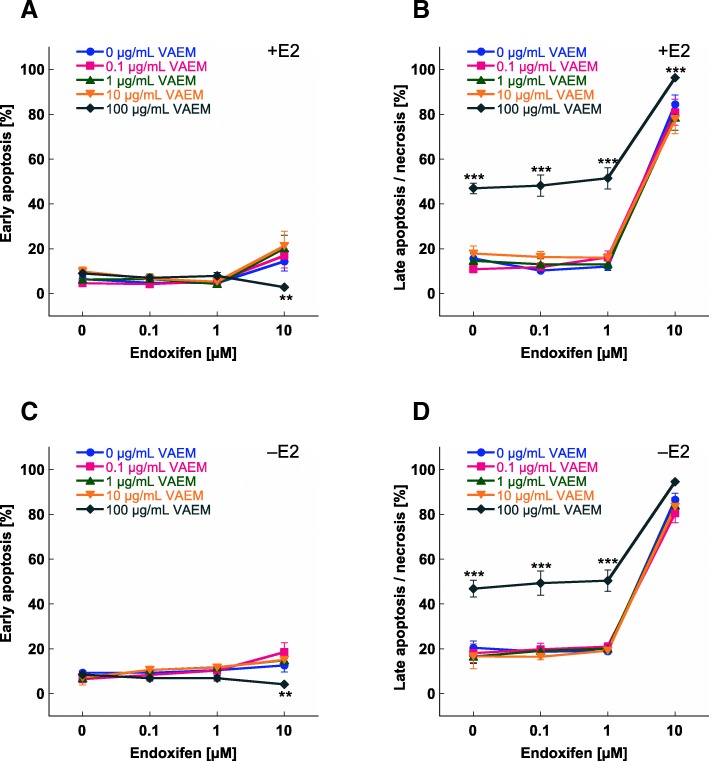
Apoptosis induction (%) in MCF-7 cells after 5 days treatment with endoxifen in combination with VAEM. Mean values (±SE) of (**a**) early apoptosis in the presence of 0.5 μM β-estradiol (E2) (**b**) late apoptosis/necrosis in the presence of 0.5 μM β-estradiol, (**c**) early apoptosis in the absence of 0.5 μM β-estradiol and (**d**) late apoptosis/necrosis in the absence of 0.5 μM β-estradiol are presented (*p < 0.05, **p < 0.01, ***p < 0.001)

Figure 4 shows the mean values of early apoptosis and late apoptosis/necrosis of MCF-7 breast carcinoma cells treated with different concentrations of endoxifen in combination with different concentrations of VAEM. In cells cultured either in the presence of estradiol or under steroid depleted conditions, only the highest VAEM concentration of 100 μg/mL significantly increased the cytotoxic effect of 0.1–10 μM endoxifen. Due to the high cytotoxicity of 10 μM endoxifen combined with 100 μg/mL VAEM, the number of early apoptotic cells was significantly reduced.

### Cell cycle

**Fig. 5:**
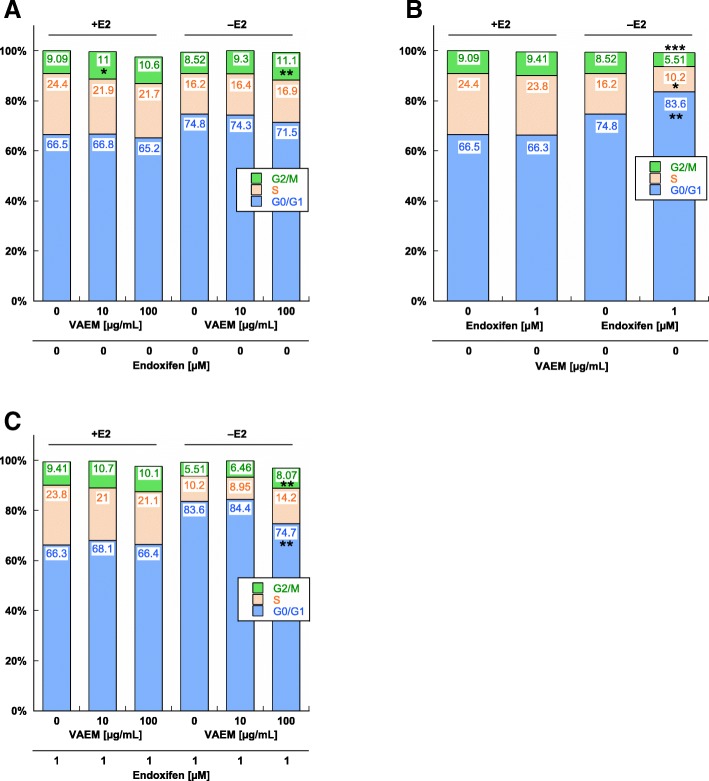
MCF-7 cell cycle analysis of cells treated with (**a**) VAEM (**b**) endoxifen and (**c**) 1 μM endoxifen and VAEM, both in presence or absence of β-estradiol (E2), respectively. Cells were harvested after 3d, fixed, stained and analyzed for DNA content by flow cytometry. The distribution and percentage of cells in G0/G1, S and G2/M phase of the cell cycle are indicated. Results are presented as mean values from six independent experiments. (SE values are omitted for clarity). Significance values are given relative to the VAEM and endoxifen untreated controls (*p < 0.05, **p < 0.01, ***p < 0.001). Discrepancies from 100% can be attributed to slight gating differences and the display of mean values

VAEM affected the cell cycle kinetics at 10 μg/mL in MCF-7 cells grown in the presence of estrogen, and at 100 μg/mL under estrogen depleted conditions by inducing a significant G2/M accumulation of 11% vs. about 9% in the appropriate control (*p* < 0.05 and *p* < 0.01, respectively) (Fig. 5a). In cells grown in the presence of estradiol, no significant effect of endoxifen on the cell cycle was observed (Fig. 5b). Under steroid depleted conditions, 1 μM endoxifen induced a significant accumulation of MCF-7 cells in the G0/G1 cell cycle phase (*p* < 0.01). The percentage of cells in G0/G1 phase was 83.6% after treatment with 1 μM endoxifen, compared to 74.8% in the control. The treatment with endoxifen also led to a significant decrease in the S (*p* < 0.05) and G2/M phase (*p* < 0.001).

In the presence of estradiol and endoxifen, VAEM showed no effect on the cell cycle. In estradiol-free MCF-7 cells simultaneously treated with endoxifen and VAEM, the G2/M arrest promoting effect of 100 μg/mL VAEM antagonized the induction of the G0/G1 arrest by endoxifen significantly (*p* < 0.01) but, as shown earlier, in combination with a profound growth reducing activity (Fig. 5c).

### CYP2D6 inhibition assays

**Fig. 6:**
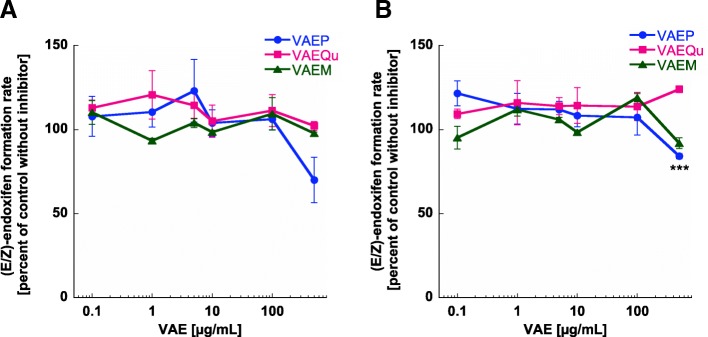
In vitro effects of mistletoe preparations (VAEP, VAEQu and VAEM) on (**a**) CYP2D6 and (**b**) CYP3A4/5 catalyzed metabolism of tamoxifen. Results are presented as mean rate values ± SE (from three independent experiments) of (E/Z)-endoxifen formation in vitro expressed as a percent of the control without inhibitor. Significance values are given relative to the negative controls without inhibitors (*p < 0.05, **p < 0.01, ***p < 0.001)

A decrease in the formation of the metabolite (dextrorphan or endoxifen) compared to vehicle control is commonly used to determine the CYP inhibition profiles. The three mistletoe preparations (VAEP, VAEQu and VAEM) were incubated separately with the CYP2D6 industry accepted probe substrate dextromethorphan (Additional file 4: Figure S1) and with the drug test substrate tamoxifen (Fig. 6a). The CYP2D6 isoform-specific inhibitor quinidine showed a significant inhibition at low concentrations for both substrates, tamoxifen (Additional file 5: Figure S2) and dextromethorphan (Additional file 6: Figure S3), thereby demonstrating that used HLMs contain the full complement of P450 enzymes and especially active CYP2D6 enzyme.

In contrast to quinidine, none of the VAE preparations showed a significant inhibition of CYP2D6 for both substrates: tamoxifen (Fig. 6a) and dextromethorphan (Additional file 4: Figure S1).

### CYP3A4/5 inhibition assays

A decrease in the formation of the metabolite (6β-hydroxytestosterone or endoxifen) compared to vehicle control is used to display the CYP inhibition profiles. The three mistletoe preparations (VAEP, VAEQu and VAEM) were incubated separately with the CYP3A4/5 industry accepted probe substrate testosterone (Additional file 7: Figure S4) and with the drug test substrate tamoxifen (Fig. 6b). CYP3A4/5 isoform-specific inhibitor ketoconazole showed a significant inhibition at low concentrations for both substrates, tamoxifen (Additional file 8: Figure S5) and testosterone (Additional file 9: Figure S6), thereby demonstrating that used HLMs contain the full complement of P450 enzymes and especially active CYP3A4/5 enzymes.

In contrast to ketoconazole, VAEM and VAEQu showed no significant inhibition of CYP3A4/5 for tamoxifen (Fig. 6b). Only VAEP at 500 μg/mL showed a statistically highly significant inhibition (*p* ≤ 0.001) of the formation of endoxifen.

At last, all VAE preparations at 100 and 500 μg/mL showed a statistically highly significant inhibition of CYP3A4/5 for testosterone as isoform-specific substrate (Additional file 7: Figure S4).

## Discussion

Our in vitro study showed that the combined treatment of MCF-7 breast cancer cells with endoxifen and VAEM did not inhibit the antiproliferative effect of the major active metabolite of tamoxifen. This finding is in line with our earlier interaction studies on VAE with several chemotherapeutic drugs and with trastuzumab, where we did not find any adverse effects of VAE [15, 16].

In our experiments, the cell growth-reducing endoxifen doses were between 10 nM and 1 μM. Endoxifen concentrations ≥10 μM led to cell death. These findings correspond with reports showing that 10–1000 nM endoxifen leads to an enhancement of ERα protein degradation by the proteasome in MCF-7 and other breast cancer cell lines. In the presence of estradiol, the transcriptional activation of an estrogen response element and the activation of estrogen-responsive endogenous genes was reduced or blocked [21]. Saji et al. reported IC_50_ values of 100 nM or 500 nM for endoxifen in MCF-7 in the absence or presence of 1 nM estradiol [22].

The proliferation of MCF-7 cells was significantly inhibited at VAEM concentrations ≥ 10 μg/mL, but at 100 μg/mL, no complete inhibition of cell growth was achieved. In cells treated simultaneously with endoxifen, VAEM concentrations ≥10 μg/mL led to an additive inhibitory effect at all endoxifen concentrations.

Although MCF-7 cells lack the expression of caspase 3, it has been shown that they are able to respond by apoptosis via sequential activation of caspases 9, 7 and 6 and P21 accumulation [23]. Our apoptosis experiments revealed a strong cytotoxic effect of 10 μM endoxifen. VAEM contains mistletoe lectins (ML) known to possess ribosome inactivating properties leading to the induction of apoptosis via the intrinsic pathway [24]. However, our results did not show an increased proportion of early apoptotic cells following treatment with VAEM with and without estradiol. Only 100 μg/mL VAEM significantly induced cell death. In all treatment variants, the proportion of early apoptotic cells compared to dead cells was low. We observed this also at a shorter treatment time (3 days, Additional file 10: Figure S7). The induction of the intrinsic apoptosis pathway by MLs seems to be impossible in MCF-7 cells probably due to the lack of caspase 3. Necroptosis [25], an alternative type of programmed cell death triggered through the same signals that induce apoptosis might be responsible for the cytotoxic effect of MLs. For several RIPs II the induction of caspase-independent cell death pathways, proven e.g. by using necrostatin-1, an inhibitor of necroptosis have been described [26–28]. That MCF-7 cells are able to conduct necroptosis was shown by Han et al. [29]. Whether necroptosis or another type of cell death, such as autophagic lytic cell death, as described for tamoxifen, 4-hydroxy-tamoxifen or ICI 164384 [30] is responsible for the cytotoxic effect of VAEM on MCF-7 cells remains to be investigated in further experiments.

The cell cycle analysis revealed the induction of a significant G0/G1 arrest by 1 μM endoxifen in estrogen-depleted cells but not in the presence of estrogen. Treatment of cells with VAEM alone at ≥10 μg/mL led to a small but significant G2/M arrest under both cell culture conditions. 100 μg/mL VAEM alone or in combination with all endoxifen concentrations dramatically lowered cell proliferation. We think that the cytostatic and cytotoxic activity together with a likely S/G2M blockade prevented the development of a G0/G1 arrest induced by endoxifen. We already observed the induction of a G2/M arrest by VAEM in an earlier in vitro study with trastuzumab [16].

In humans, tamoxifen is metabolized in the liver by members of the cytochrome P450 enzyme family. The primary metabolic route leads from tamoxifen via CYP3A catalyzed N-demethylation to N-desmethyl tamoxifen and via CYP2D6 mediated hydroxylation to endoxifen [31]. The CYP2D6 gene is highly polymorphic. Based on genotype combinations encoding for varying enzyme phenotypes, patients have been classified as poor, intermediate, extensive, or ultra-rapid metabolizers [32, 33]. The plasma concentration of the most potent tamoxifen metabolite endoxifen depends, among others, on the CYP2D6 metabolizer phenotype [34]. The metabolic activity of CYP2D6 was suggested to be associated with the long-term outcomes in breast cancer, although the studies are inconsistent [35]. Given that cytochrome P450 enzymes are critical for phase I drug metabolism, other medications might interfere with tamoxifen metabolism and thereby increase the risk of cancer recurrence. This is assumed for several antidepressants, especially some selective serotonin reuptake inhibitors (SSRIs) prescribed to alleviate disease-related problems and side effects of the anti-hormonal treatment like anxiety, depression or hot flashes [36, 37]. Recommendations exist to avoid the prescription of antidepressants most likely to inhibit CYP2D6 [36, 38, 39].

For the CYP3A4/5 and CYP2D6 inhibition assays in this study, we used 50-donor mixed gender pooled human liver microsomes rather than recombinant isoenzymes, given that microsomes contain the full set of CYP450 enzymes and thus are closer to the in vivo situation [40]. The significant inter-individual variability of CYP2D6 activity due to genetic polymorphisms was mitigated by the use of pooled microsomes from 50 donors [41]. With exception of a significant inhibition of CYP3A4/5 by 500 μg/mL of VAEP, all other VAE preparations showed no significant inhibition of CYP3A4/5 and CYP2D6 at all test concentrations (Fig. 6). Thus, we conclude that mistletoe extracts do not interfere with CYP450 (3A4/5 and 2D6) in their ability to metabolize tamoxifen.

A possible inhibition of CYP3A4 by mistletoe preparations has been investigated in an in vitro study earlier [42]. Our data confirm these findings and suggest that clinically relevant systemic interactions with CYP3A4 and CYP2D6 are unlikely.

The positive effects of an adjuvant treatment with mistletoe preparations on the quality of life and cancer-related fatigue have been reported in several clinical studies [11, 43]. Our investigations of mistletoe interactions with CYP3A4/5 and CYP2D6, secure recommendations for the use of mistletoe extracts to treat cancer related-fatigue in breast cancer patients receiving tamoxifen.

Given the prevalent use of CAM by cancer patients, there is an increasing need to assess the risk of combining CAM with conventional medications. Mistletoe belongs to the medicinal herbs that are widely used as supportive care during standard anticancer therapy. The present in vitro study contributes to the knowledge of possible herb-drug interactions and supports the assessment of breast cancer patient’s safety hazard by potential adverse reactions from the combination of *Viscum album* preparations and tamoxifen.

## Conclusions

We could demonstrate in vitro that clinically relevant doses of an aqueous, fermented mistletoe preparation did not inhibit the cytostatic and cytotoxic activity of (E/Z)-endoxifen, an active metabolite of tamoxifen. Higher doses showed additive effects. VAE preparations did not show inhibition of CYP3A4/5 and CYP2D6.

These findings further substantiate the knowledge on the safety of breast cancer patients receiving tamoxifen concomitantly with mistletoe preparations.

## Additional files

Additional file 1: **Table S1.** Optimized MS/MS parameters in ESI positive mode for analytes (S: substrate and M: metabolite) and corresponding IS. (PDF 14 kb)

Additional file 2: Data_proliferation_apoptosis_cell_cycle. (PDF 219 kb)

Additional file 3: Data_CYP2D6_and_CYP3A4-5_inhibition assays. (PDF 105 kb)

Additional file 4: **Figure S1.** Dextromethorphan in vitro inhibition profiles of CYP2D6 by mistletoe preparations (VAEP, VAEQu, and VAEM). Results are presented as mean rate values ± SE (from three independent experiments) of dextrorphan formation in vitro expressed as a percent of the control without inhibitor. Significance values are given relative to the negative controls without inhibitors (*p<0.05, **p < 0.01, ***p < 0.001). (PDF 36 kb)

Additional file 5: **Figure S2.** Tamoxifen in vitro inhibition profiles of CYP2D6 by reference inhibitor: Quinidine (0.1-1-2-5-10-100 μM). Results are presented as mean rate values ± SE (from three independent experiments) of (E/Z)-endoxifen formation in vitro expressed as a percent of the control without inhibitor. (PDF 27 kb)

Additional file 6: **Figure S3.** Dextromethorphan in vitro inhibition profiles of CYP2D6 by reference inhibitor: Quinidine (0.1-1-2-5-10-100 μM). Results are presented as mean rate values ± SE (from three independent experiments) of dextrorphan formation in vitro expressed as a percent of the control without inhibitor. (PDF 26 kb)

Additional file 7: **Figure S4.** Testosterone in vitro inhibition profiles of CYP3A4/5 (VAEP, VAEQu, VAEM). Results are presented as mean rate values ± SE (from three independent experiments) of 6β-hydroxytestosterone formation in vitro expressed as a percent of the control without inhibitor. (PDF 40 kb)

Additional file 8: **Figure S5.** Tamoxifen in vitro inhibition profile of CYP3A4/5 by reference inhibitor: Ketoconazole (0.1-1-2-5-10-100 μM). Results are presented as mean rate values ± SE (from three independent experiments) of (E/Z)-endoxifen formation in vitro expressed as a percent of the control without inhibitor. (PDF 27 kb)

Additional file 9: **Figure S6.** Testosterone in vitro inhibition profile of CYP3A4/5 by reference inhibitor: Ketoconazole (0.1-1-2-5-10-100 μM). Results are presented as mean rate values ± SE (from three independent experiments) of 6β-hydroxytestosterone formation in vitro expressed as a percent of the control without inhibitor. (PDF 31 kb)

Additional file 10: **Figure S7.** Apoptosis induction (%) in MCF-7 cells after 3d treatment with endoxifen in combination with VAEM. Mean values (±SE) of (A) early apoptosis in the presence of 0.5 μM β-estradiol (E2) (B) late apoptosis/necrosis in the presence of 0.5 μM β-estradiol, (C) early apoptosis in the absence of 0.5 μM β-estradiol and (D) late apoptosis/necrosis in the absence of 0.5 μM β-estradiol are presented (*p < 0.05, **p < 0.01, ***p < 0.001). (PDF 53 kb)

## Abbreviations

CAM: Complementary and Alternative Medicine
Cyp2D6: Cytochrome P450 2D6
Cyp3A4/5: Cytochrome P450 3A4/5
E2: β-estradiol
ER: Estrogen receptor
FCS: Fetal calf serum
HDIs: Herb-drug interactions
HLMs: Human liver microsomes
IS: Internal standard
ML: Mistletoe lectin
VAE: *Viscum album* extracts
VAEM: VAE from host tree *Malus domestic*
VAEP: VAE from host tree *Pinus sylvestris*
VAEQu: VAE from host tree *Quercus robur petraea*
