# Supplementary material for: Absence of herb-drug interactions of mistletoe with the tamoxifen metabolite (E/Z)-endoxifen and cytochrome P450 3A4/5 and 2D6 in vitro
Source: BMC Complement Altern Med. 2019 Jan 18;19:23. doi: 10.1186/s12906-019-2439-2 (PMC6339413; doi:10.1186/s12906-019-2439-2)
Supplement: Supplementary file 1 — Table S1. Optimized MS/MS parameters in ESI positive mode for analytes (S: substrate and M: metabolite) and corresponding IS. (PDF 14 kb) [file 12906_2019_2439_MOESM1_ESM.pdf]

Table S1 Optimized MS/MS parameters in ESI positive mode for analytes (S: substrate and M: metabolite) and corresponding IS

**Substrate (S)**<br>**Metabolite (M)**<br>**Internal standard (IS)**	**MRM transitions**	**Fragmentor voltage (V)**	**Collision energy (eV)**
S: Tamoxifen	372.23 > 72.2	142	26
M: Endoxifen	374.21 > 58.2	147	18
IS: Endoxifen-d5	379.25 > 58.2	147	22
S: Testosterone	289.22 > 97.1	152	22
M: 6β-hydroxytestosterone	305.21 > 77.1	127	82
IS: Testosterone-d3	292.24 > 97.1	132	22
S: Dextromethorphan	272.20 > 171.1	127	42
M: Dextrorphan	258.19 > 157.1	142	42
IS: Dextrorphan-d3	261.21 > 157.1	142	42
