# Supplementary material for: Absence of herb-drug interactions of mistletoe with the tamoxifen metabolite (E/Z)-endoxifen and cytochrome P450 3A4/5 and 2D6 in vitro
Source: BMC Complement Altern Med. 2019 Jan 18;19:23. doi: 10.1186/s12906-019-2439-2 (PMC6339413; doi:10.1186/s12906-019-2439-2)
Supplement: Supplementary file 2 — Data_proliferation_apoptosis_cell_cycle. (PDF 219 kb) [file 12906_2019_2439_MOESM2_ESM.pdf]

WST-1 without estradiol (treatment 7d)

date	Endoxifen_uM	Endoxifen_uM	Endoxifen_M	Estradiol	Iscador ug/ml	viab%control
150128	0	0.00E+00	0.00E+00	no	0	107.06
140204	0	0.00E+00	0.00E+00	no	0	107.74
150211	0	0.00E+00	0.00E+00	no	0	100.52
150128	0.0001	1.00E-04	1.00E-10	no	0	102.88
140204	0.0001	1.00E-04	1.00E-10	no	0	106.30
150211	0.0001	1.00E-04	1.00E-10	no	0	100.71
150128	0.01	1.00E-02	1.00E-08	no	0	77.02
140204	0.01	1.00E-02	1.00E-08	no	0	92.49
150211	0.01	1.00E-02	1.00E-08	no	0	85.82
150128	1	1.00E+00	1.00E-06	no	0	65.67
140204	1	1.00E+00	1.00E-06	no	0	77.99
150211	1	1.00E+00	1.00E-06	no	0	71.50
150128	100	1.00E+02	1.00E-04	no	0	0.65
140204	100	1.00E+02	1.00E-04	no	0	1.53
150211	100	1.00E+02	1.00E-04	no	0	1.70
150128	0	0.00E+00	0.00E+00	no	0.1	102.07
140204	0	0.00E+00	0.00E+00	no	0.1	103.29
150211	0	0.00E+00	0.00E+00	no	0.1	95.89
150128	0.0001	1.00E-04	1.00E-10	no	0.1	98.31
140204	0.0001	1.00E-04	1.00E-10	no	0.1	110.80
150211	0.0001	1.00E-04	1.00E-10	no	0.1	97.21
150128	0.01	1.00E-02	1.00E-08	no	0.1	81.93
140204	0.01	1.00E-02	1.00E-08	no	0.1	93.10
150211	0.01	1.00E-02	1.00E-08	no	0.1	82.80
150128	1	1.00E+00	1.00E-06	no	0.1	72.69
140204	1	1.00E+00	1.00E-06	no	0.1	85.73
150211	1	1.00E+00	1.00E-06	no	0.1	77.98
150128	100	1.00E+02	1.00E-04	no	0.1	0.12

140204	100	1.00E+02	1.00E-04	no	0.1	0.88
150211	100	1.00E+02	1.00E-04	no	0.1	0.85
150128	0	0.00E+00	0.00E+00	no	1	105.49
140204	0	0.00E+00	0.00E+00	no	1	100.37
150211	0	0.00E+00	0.00E+00	no	1	92.72
150128	0.0001	1.00E-04	1.00E-10	no	1	98.62
140204	0.0001	1.00E-04	1.00E-10	no	1	104.03
150211	0.0001	1.00E-04	1.00E-10	no	1	94.23
150128	0.01	1.00E-02	1.00E-08	no	1	77.64
140204	0.01	1.00E-02	1.00E-08	no	1	94.86
150211	0.01	1.00E-02	1.00E-08	no	1	81.33
150128	1	1.00E+00	1.00E-06	no	1	70.08
140204	1	1.00E+00	1.00E-06	no	1	84.48
150211	1	1.00E+00	1.00E-06	no	1	70.89
150128	100	1.00E+02	1.00E-04	no	1	-0.15
140204	100	1.00E+02	1.00E-04	no	1	0.74
150211	100	1.00E+02	1.00E-04	no	1	1.04
150128	0	0.00E+00	0.00E+00	no	10	91.94
140204	0	0.00E+00	0.00E+00	no	10	85.82
150211	0	0.00E+00	0.00E+00	no	10	82.89
150128	0.0001	1.00E-04	1.00E-10	no	10	78.60
140204	0.0001	1.00E-04	1.00E-10	no	10	87.91
150211	0.0001	1.00E-04	1.00E-10	no	10	83.13
150128	0.01	1.00E-02	1.00E-08	no	10	70.85
140204	0.01	1.00E-02	1.00E-08	no	10	75.16
150211	0.01	1.00E-02	1.00E-08	no	10	68.71
150128	1	1.00E+00	1.00E-06	no	10	61.83
140204	1	1.00E+00	1.00E-06	no	10	67.52
150211	1	1.00E+00	1.00E-06	no	10	59.59
150128	100	1.00E+02	1.00E-04	no	10	0.04
140204	100	1.00E+02	1.00E-04	no	10	0.65

150211	100	1.00E+02	1.00E-04	no	10	0.76
150128	0	0.00E+00	0.00E+00	no	100	11.78
140204	0	0.00E+00	0.00E+00	no	100	15.38
150211	0	0.00E+00	0.00E+00	no	100	17.96
150128	0.0001	1.00E-04	1.00E-10	no	100	12.01
140204	0.0001	1.00E-04	1.00E-10	no	100	14.97
150211	0.0001	1.00E-04	1.00E-10	no	100	18.15
150128	0.01	1.00E-02	1.00E-08	no	100	11.28
140204	0.01	1.00E-02	1.00E-08	no	100	13.81
150211	0.01	1.00E-02	1.00E-08	no	100	17.25
150128	1	1.00E+00	1.00E-06	no	100	7.36
140204	1	1.00E+00	1.00E-06	no	100	8.67
150211	1	1.00E+00	1.00E-06	no	100	11.29
150128	100	1.00E+02	1.00E-04	no	100	-1.19
140204	100	1.00E+02	1.00E-04	no	100	-0.56
150211	100	1.00E+02	1.00E-04	no	100	-0.57

WST-1 with estradiol (treatment 7d)

date	Endoxifen_uM	Endoxifen_uM	Endoxifen_M	Estradiol	Iscador ug/ml	viab%control
141106	100	1.00E+02	1.00E-04	yes	0	2.1
141113	100	1.00E+02	1.00E-04	yes	0	1.3
141120	100	1.00E+02	1.00E-04	yes	0	1.5
150218	100	1.00E+02	1.00E-04	yes	0	2.3
141106	1	1.00E+00	1.00E-06	yes	0	167.4
141113	1	1.00E+00	1.00E-06	yes	0	142.0
141120	1	1.00E+00	1.00E-06	yes	0	164.3
150218	1	1.00E+00	1.00E-06	yes	0	128.3
141106	0.01	1.00E-02	1.00E-08	yes	0	196.0
141113	0.01	1.00E-02	1.00E-08	yes	0	196.8
141120	0.01	1.00E-02	1.00E-08	yes	0	201.6
150218	0.01	1.00E-02	1.00E-08	yes	0	161.2
141113	0.0001	1.00E-04	1.00E-10	yes	0	195.1
141120	0.0001	1.00E-04	1.00E-10	yes	0	207.5
150218	0.0001	1.00E-04	1.00E-10	yes	0	156.1
141106	0	0.00E+00	0.00E+00	yes	0	196.1
141113	0	0.00E+00	0.00E+00	yes	0	185.3
141120	0	0.00E+00	0.00E+00	yes	0	207.4
150218	0	0.00E+00	0.00E+00	yes	0	155.9
141106	100	1.00E+02	1.00E-04	yes	0.1	1.6
141113	100	1.00E+02	1.00E-04	yes	0.1	0.4
141120	100	1.00E+02	1.00E-04	yes	0.1	1.3
150218	100	1.00E+02	1.00E-04	yes	0.1	1.6
141106	1	1.00E+00	1.00E-06	yes	0.1	165.1
141113	1	1.00E+00	1.00E-06	yes	0.1	170.4
141120	1	1.00E+00	1.00E-06	yes	0.1	185.9
150218	1	1.00E+00	1.00E-06	yes	0.1	138.8
141106	0.01	1.00E-02	1.00E-08	yes	0.1	195.2

141113	0.01	1.00E-02	1.00E-08	yes	0.1	204.5
141120	0.01	1.00E-02	1.00E-08	yes	0.1	200.0
150218	0.01	1.00E-02	1.00E-08	yes	0.1	158.4
141113	0.0001	1.00E-04	1.00E-10	yes	0.1	190.6
141120	0.0001	1.00E-04	1.00E-10	yes	0.1	192.1
150218	0.0001	1.00E-04	1.00E-10	yes	0.1	156.3
141106	0	0.00E+00	0.00E+00	yes	0.1	187.7
141113	0	0.00E+00	0.00E+00	yes	0.1	201.0
141120	0	0.00E+00	0.00E+00	yes	0.1	213.9
150218	0	0.00E+00	0.00E+00	yes	0.1	161.5
141106	100	1.00E+02	1.00E-04	yes	1	1.4
141113	100	1.00E+02	1.00E-04	yes	1	0.1
141120	100	1.00E+02	1.00E-04	yes	1	1.1
150218	100	1.00E+02	1.00E-04	yes	1	1.1
141106	1	1.00E+00	1.00E-06	yes	1	164.2
141113	1	1.00E+00	1.00E-06	yes	1	158.8
141120	1	1.00E+00	1.00E-06	yes	1	168.1
150218	1	1.00E+00	1.00E-06	yes	1	121.7
141106	0.01	1.00E-02	1.00E-08	yes	1	187.2
141113	0.01	1.00E-02	1.00E-08	yes	1	192.4
141120	0.01	1.00E-02	1.00E-08	yes	1	201.1
150218	0.01	1.00E-02	1.00E-08	yes	1	156.6
141113	0.0001	1.00E-04	1.00E-10	yes	1	200.1
141120	0.0001	1.00E-04	1.00E-10	yes	1	207.1
150218	0.0001	1.00E-04	1.00E-10	yes	1	158.1
141106	0	0.00E+00	0.00E+00	yes	1	192.1
141113	0	0.00E+00	0.00E+00	yes	1	189.1
141120	0	0.00E+00	0.00E+00	yes	1	197.3
150218	0	0.00E+00	0.00E+00	yes	1	150.4
141106	100	1.00E+02	1.00E-04	yes	10	1.1
141113	100	1.00E+02	1.00E-04	yes	10	0.5

141120	100	1.00E+02	1.00E-04	yes	10	-1.3
150218	100	1.00E+02	1.00E-04	yes	10	1.0
141106	1	1.00E+00	1.00E-06	yes	10	128.5
141113	1	1.00E+00	1.00E-06	yes	10	143.5
141120	1	1.00E+00	1.00E-06	yes	10	126.0
150218	1	1.00E+00	1.00E-06	yes	10	107.2
141106	0.01	1.00E-02	1.00E-08	yes	10	149.4
141113	0.01	1.00E-02	1.00E-08	yes	10	171.5
141120	0.01	1.00E-02	1.00E-08	yes	10	163.0
150218	0.01	1.00E-02	1.00E-08	yes	10	132.4
141113	0.0001	1.00E-04	1.00E-10	yes	10	151.7
141120	0.0001	1.00E-04	1.00E-10	yes	10	162.0
150218	0.0001	1.00E-04	1.00E-10	yes	10	123.4
141106	0	0.00E+00	0.00E+00	yes	10	165.0
141113	0	0.00E+00	0.00E+00	yes	10	168.0
141120	0	0.00E+00	0.00E+00	yes	10	163.7
150218	0	0.00E+00	0.00E+00	yes	10	134.6
150218	100	1.00E+02	1.00E-04	yes	100	0.0
150218	100	1.00E+01	1.00E-05	yes	100	-0.4
150218	100	1.00E+01	1.00E-05	yes	100	-0.1
150218	100	1.00E+01	1.00E-05	yes	100	-1.1
150218	1	1.00E+00	1.00E-06	yes	100	16.2
141106	1	1.00E+00	1.00E-06	yes	100	5.1
141120	1	1.00E+00	1.00E-06	yes	100	3.7
150218	1	1.00E+00	1.00E-06	yes	100	4.3
171025	0.01	1.00E-02	1.00E-08	yes	100	27.6
171025	0.01	1.00E-02	1.00E-08	yes	100	8.5
171025	0.01	1.00E-02	1.00E-08	yes	100	7.9
171025	0.01	1.00E-02	1.00E-08	yes	100	8.1
171025	0.0001	1.00E-04	1.00E-10	yes	100	27.7
171025	0.0001	1.00E-04	1.00E-10	yes	100	7.0

171025	0.0001	1.00E-04	1.00E-10	yes	100	6.1
171025	0.0001	1.00E-04	1.00E-10	yes	100	6.0
171025	0	0.00E+00	0.00E+00	yes	100	49.1
171025	0	0.00E+00	0.00E+00	yes	100	16.7
171025	0	0.00E+00	0.00E+00	yes	100	26.1
171025	0	0.00E+00	0.00E+00	yes	100	9.3
171025	0	0.00E+00	0.00E+00	yes	100	7.4
171025	0	0.00E+00	0.00E+00	yes	100	8.6

Apoptosis without estradiol

date	Iscador ug/ml	Konz Endox uM	Konz Endox uM	Konz Endox M	time (d)	Estradiol	viable	EA	LA/N
150511	0	0	0.00E+00	0.00E+00	3	no	83.7	4.27	12
150511	0.1	0	0.00E+00	0.00E+00	3	no	84	4.31	11.66
150511	1	0	0.00E+00	0.00E+00	3	no	80.8	6.11	13.14
150511	10	0	0.00E+00	0.00E+00	3	no	86	3.52	10.51
150511	0	0.1	1.00E-01	1.00E-07	3	no	80.3	5.35	14.31
150511	0.1	0.1	1.00E-01	1.00E-07	3	no	78.7	5.78	15.45
150511	1	0.1	1.00E-01	1.00E-07	3	no	76.2	7.3	16.55
150511	10	0.1	1.00E-01	1.00E-07	3	no	77.7	5.07	17.21
150511	0	1	1.00E+00	1.00E-06	3	no	78	7.3	14.73
150511	0.1	1	1.00E+00	1.00E-06	3	no	79.2	4.89	15.91
150511	1	1	1.00E+00	1.00E-06	3	no	76.3	5.74	17.97
150511	10	1	1.00E+00	1.00E-06	3	no	77.1	6.28	16.61
150511	0	10	1.00E+01	1.00E-05	3	no	32.6	7.18	60.2
150511	0.1	10	1.00E+01	1.00E-05	3	no	25.8	6.73	67.5
150511	1	10	1.00E+01	1.00E-05	3	no	18.9	9.36	71.82
150511	10	10	1.00E+01	1.00E-05	3	no	26.6	11.4	62.01
150518	0	0	0.00E+00	0.00E+00	3	no	84.8	7.86	7.34
150518	0.1	0	0.00E+00	0.00E+00	3	no	86.6	6.63	6.81
150518	1	0	0.00E+00	0.00E+00	3	no	85.1	8.36	6.55
150518	10	0	0.00E+00	0.00E+00	3	no	88.9	3.67	7.4
150518	0	0.1	1.00E-01	1.00E-07	3	no	86.4	5.87	7.68
150518	0.1	0.1	1.00E-01	1.00E-07	3	no	85.9	5.47	8.68
150518	1	0.1	1.00E-01	1.00E-07	3	no	86.9	6.35	6.72
150518	10	0.1	1.00E-01	1.00E-07	3	no	85.7	5.36	8.9
150518	0	1	1.00E+00	1.00E-06	3	no	81.4	7.29	11.35
150518	0.1	1	1.00E+00	1.00E-06	3	no	84.5	7.09	8.46
150518	1	1	1.00E+00	1.00E-06	3	no	87	5.95	7.08
150518	10	1	1.00E+00	1.00E-06	3	no	82.8	9.17	8.06

150518	0	10	1.00E+01	1.00E-05	3	no	23	9.94	67.05
150518	0.1	10	1.00E+01	1.00E-05	3	no	23.9	11.9	64.21
150518	1	10	1.00E+01	1.00E-05	3	no	19.7	8.71	71.57
150518	10	10	1.00E+01	1.00E-05	3	no	26	7.63	66.44
150601	0	0	0.00E+00	0.00E+00	3	no	70.8	3.24	26.02
150601	0.1	0	0.00E+00	0.00E+00	3	no	71.5	5.47	23
150601	1	0	0.00E+00	0.00E+00	3	no	70.9	3.64	25.43
150601	10	0	0.00E+00	0.00E+00	3	no	68.8	2.49	28.72
150601	0	0.1	1.00E-01	1.00E-07	3	no	74.2	4.37	21.36
150601	0.1	0.1	1.00E-01	1.00E-07	3	no	70.9	5.25	23.87
150601	1	0.1	1.00E-01	1.00E-07	3	no	74.1	4.41	21.54
150601	10	0.1	1.00E-01	1.00E-07	3	no	70.3	3.59	26.14
150601	0	1	1.00E+00	1.00E-06	3	no	73.5	3.28	23.16
150601	0.1	1	1.00E+00	1.00E-06	3	no	69.9	6.24	23.9
150601	1	1	1.00E+00	1.00E-06	3	no	71.7	5.9	22.38
150601	10	1	1.00E+00	1.00E-06	3	no	73	6.27	20.8
150601	0	10	1.00E+01	1.00E-05	3	no	11.1	5.53	83.4
150601	0.1	10	1.00E+01	1.00E-05	3	no	12.4	7.41	80.17
150601	1	10	1.00E+01	1.00E-05	3	no	12.2	4.2	83.6
150601	10	10	1.00E+01	1.00E-05	3	no	10.9	4.58	84.6
150513	0	0	0.00E+00	0.00E+00	5	no	78.4	8.9	12.7
150513	0.1	0	0.00E+00	0.00E+00	5	no	82.9	6.7	10.4
150513	1	0	0.00E+00	0.00E+00	5	no	83.6	5.9	10.6
150513	10	0	0.00E+00	0.00E+00	5	no	88.0	5.6	6.4
150513	0	0.1	1.00E-01	1.00E-07	5	no	75.1	9.6	15.3
150513	0.1	0.1	1.00E-01	1.00E-07	5	no	77.4	7.7	14.8
150513	1	0.1	1.00E-01	1.00E-07	5	no	73.5	9.0	17.5
150513	10	0.1	1.00E-01	1.00E-07	5	no	73.2	10.7	16.0
150513	0	1	1.00E+00	1.00E-06	5	no	73.0	10.4	16.7
150513	0.1	1	1.00E+00	1.00E-06	5	no	72.9	9.0	18.2
150513	1	1	1.00E+00	1.00E-06	5	no	72.6	9.3	18.1

150513	10	1	1.00E+00	1.00E-06	5	no	71.8	11.6	16.5
150513	0	10	1.00E+01	1.00E-05	5	no	1.2	11.0	87.7
150513	0.1	10	1.00E+01	1.00E-05	5	no	0.9	17.0	82.1
150513	1	10	1.00E+01	1.00E-05	5	no	0.6	13.3	86.0
150513	10	10	1.00E+01	1.00E-05	5	no	1.1	16.8	82.2
150520	0	0	0.00E+00	0.00E+00	5	no	72.5	9.8	17.7
150520	0.1	0	0.00E+00	0.00E+00	5	no	68.2	6.9	24.9
150520	1	0	0.00E+00	0.00E+00	5	no	71.7	9.5	18.8
150520	10	0	0.00E+00	0.00E+00	5	no	60.9	13.3	25.8
150520	0	0.1	1.00E-01	1.00E-07	5	no	69.4	10.8	19.7
150520	0.1	0.1	1.00E-01	1.00E-07	5	no	64.9	11.1	24.0
150520	1	0.1	1.00E-01	1.00E-07	5	no	68.8	10.9	20.3
150520	10	0.1	1.00E-01	1.00E-07	5	no	69.6	11.4	19.0
150520	0	1	1.00E+00	1.00E-06	5	no	67.1	12.2	20.7
150520	0.1	1	1.00E+00	1.00E-06	5	no	62.8	12.9	24.3
150520	1	1	1.00E+00	1.00E-06	5	no	63.0	13.1	23.9
150520	10	1	1.00E+00	1.00E-06	5	no	66.9	12.6	20.5
150520	0	10	1.00E+01	1.00E-05	5	no	0.7	8.5	90.8
150520	0.1	10	1.00E+01	1.00E-05	5	no	0.8	12.2	87.0
150520	1	10	1.00E+01	1.00E-05	5	no	0.7	10.4	88.9
150520	10	10	1.00E+01	1.00E-05	5	no	0.8	12.0	87.2
160603	0	0	0.00E+00	0.00E+00	5	no	73	5.07	21.9
160603	0.1	0	0.00E+00	0.00E+00	5	no	74.9	5.94	19.1
160603	1	0	0.00E+00	0.00E+00	5	no	74.4	5.32	20.3
160603	10	0	0.00E+00	0.00E+00	5	no	79.6	2.53	17.8
160603	0	0.1	1.00E-01	1.00E-07	5	no	71.2	7.42	21.4
160603	0.1	0.1	1.00E-01	1.00E-07	5	no	73.2	6.25	20.6
160603	1	0.1	1.00E-01	1.00E-07	5	no	68.7	11.7	19.6
160603	10	0.1	1.00E-01	1.00E-07	5	no	76	9.61	14.4
160603	0	1	1.00E+00	1.00E-06	5	no	71.5	9	19.5
160603	0.1	1	1.00E+00	1.00E-06	5	no	70.4	9.06	20.6

160603	1	1	1.00E+00	1.00E-06	5	no	68.9	12.8	18.3
160603	10	1	1.00E+00	1.00E-06	5	no	68.4	10.9	20.7
160603	0	10	1.00E+01	1.00E-05	5	no	0.479	18.4	81.2
160603	0.1	10	1.00E+01	1.00E-05	5	no	0.997	26.5	72.5
160603	1	10	1.00E+01	1.00E-05	5	no	1.57	21.2	77.2
160603	10	10	1.00E+01	1.00E-05	5	no	2.77	16.5	80.7
150520	100	0	0.00E+00	0.00E+00	5	no	46.6	11.5	41.9
160603	100	0	0.00E+00	0.00E+00	5	no	36.5	9.4	54.2
171018	0	0	0.00E+00	0.00E+00	5	no	54.2	12	33.8
171018	100	0	0.00E+00	0.00E+00	5	no	38.2	5.2	56.7
171018	100	0.1	1.00E-01	1.00E-07	5	no	35.9	4.5	59.6
171018	100	1	1.00E+00	1.00E-06	5	no	37.6	4.4	58
171018	100	10	1.00E+01	1.00E-05	5	no	0.7	3.7	95.6
171018	0	0	0.00E+00	0.00E+00	5	no	71.7	11	17.4
171018	100	0	0.00E+00	0.00E+00	5	no	55.6	7.9	36.5
171018	100	0.1	1.00E-01	1.00E-07	5	no	50.9	7.6	41.4
171018	100	1	1.00E+00	1.00E-06	5	no	50.2	8.3	41.6
171018	100	10	1.00E+01	1.00E-05	5	no	1.5	5.1	93.3
171018	0	0	0.00E+00	0.00E+00	5	no	70.9	9.6	19.5
171018	100	0	0.00E+00	0.00E+00	5	no	46.1	8.8	45
171018	100	0.1	1.00E-01	1.00E-07	5	no	44.2	8.7	47
171018	100	1	1.00E+00	1.00E-06	5	no	40.2	8.1	51.8
171018	100	10	1.00E+01	1.00E-05	5	no	1.3	3.9	94.8

Apoptosis with estradiol

Date	Iscador ug/ml	Endox uM	Endox uM	Endox M	time (d)	Estradiol	viable	EA	LA/N
150511	0	0	0.00E+00	0.00E+00	3	yes	86	5.68	8.28
150511	0.1	0	0.00E+00	0.00E+00	3	yes	84.8	4.58	10.58
150511	1	0	0.00E+00	0.00E+00	3	yes	82	7.53	10.45
150511	10	0	0.00E+00	0.00E+00	3	yes	77.2	8.26	14.53
150511	0	0.1	1.00E-01	1.00E-07	3	yes	82.6	7.19	10.17
150511	0.1	0.1	1.00E-01	1.00E-07	3	yes	86.7	3.66	9.58
150511	1	0.1	1.00E-01	1.00E-07	3	yes	82.3	5.33	12.34
150511	10	0.1	1.00E-01	1.00E-07	3	yes	82	6.4	11.62
150511	0	1	1.00E+00	1.00E-06	3	yes	83.7	7.11	9.18
150511	0.1	1	1.00E+00	1.00E-06	3	yes	83.6	4.09	12.34
150511	1	1	1.00E+00	1.00E-06	3	yes	87.4	3.44	9.19
150511	10	1	1.00E+00	1.00E-06	3	yes	81.4	7.32	11.31
150511	0	10	1.00E+01	1.00E-05	3	yes	24.7	7.62	67.7
150511	0.1	10	1.00E+01	1.00E-05	3	yes	21.3	8.59	70.11
150511	1	10	1.00E+01	1.00E-05	3	yes	21.2	8.55	70.2
150511	10	10	1.00E+01	1.00E-05	3	yes	19.3	9.12	71.58
150518	0	0	0.00E+00	0.00E+00	3	yes	84.8	7.86	7.34
150518	0.1	0	0.00E+00	0.00E+00	3	yes	86.6	6.63	6.81
150518	1	0	0.00E+00	0.00E+00	3	yes	85.1	8.36	6.55
150518	10	0	0.00E+00	0.00E+00	3	yes	88.9	3.67	7.4
150518	0	0.1	1.00E-01	1.00E-07	3	yes	86.4	5.87	7.68
150518	0.1	0.1	1.00E-01	1.00E-07	3	yes	85.9	5.47	8.68
150518	1	0.1	1.00E-01	1.00E-07	3	yes	86.9	6.35	6.72
150518	10	0.1	1.00E-01	1.00E-07	3	yes	85.7	5.36	8.9
150518	0	1	1.00E+00	1.00E-06	3	yes	81.4	7.29	11.35
150518	0.1	1	1.00E+00	1.00E-06	3	yes	84.5	7.09	8.46
150518	1	1	1.00E+00	1.00E-06	3	yes	87	5.95	7.08
150518	10	1	1.00E+00	1.00E-06	3	yes	82.8	9.17	8.06

150518	0	10	1.00E+01	1.00E-05	3	yes	23	9.94	67.05
150518	0.1	10	1.00E+01	1.00E-05	3	yes	23.9	11.9	64.21
150518	1	10	1.00E+01	1.00E-05	3	yes	19.7	8.71	71.57
150518	10	10	1.00E+01	1.00E-05	3	yes	26	7.63	66.44
150601	0	0	0.00E+00	0.00E+00	3	yes	78.6	3.28	18.09
150601	0.1	0	0.00E+00	0.00E+00	3	yes	68.1	4.99	26.96
150601	1	0	0.00E+00	0.00E+00	3	yes	80.1	2.27	17.72
150601	10	0	0.00E+00	0.00E+00	3	yes	78.3	3.22	18.53
150601	0	0.1	1.00E-01	1.00E-07	3	yes	71.4	4.02	24.57
150601	0.1	0.1	1.00E-01	1.00E-07	3	yes	76.7	2.77	20.56
150601	1	0.1	1.00E-01	1.00E-07	3	yes	77.4	2.16	20.39
150601	10	0.1	1.00E-01	1.00E-07	3	yes	79.6	2.63	17.83
150601	0	1	1.00E+00	1.00E-06	3	yes	82.8	3.06	14.09
150601	0.1	1	1.00E+00	1.00E-06	3	yes	66.8	4.21	28.91
150601	1	1	1.00E+00	1.00E-06	3	yes	76.1	2.73	21.18
150601	10	1	1.00E+00	1.00E-06	3	yes	78.3	2.97	18.72
150601	0	10	1.00E+01	1.00E-05	3	yes	19.3	8.36	72.3
150601	0.1	10	1.00E+01	1.00E-05	3	yes	12.6	7.19	80.24
150601	1	10	1.00E+01	1.00E-05	3	yes	16.6	9.02	74.36
150601	10	10	1.00E+01	1.00E-05	3	yes	15.5	6.87	77.56
150513	0	0	0.00E+00	0.00E+00	5	yes	84.0	7.3	8.6
150513	0.1	0	0.00E+00	0.00E+00	5	yes	85.1	4.5	10.4
150513	1	0	0.00E+00	0.00E+00	5	yes	86.6	5.2	8.2
150513	10	0	0.00E+00	0.00E+00	5	yes	74.7	11.9	13.4
150513	0	0.1	1.00E-01	1.00E-07	5	yes	84.9	6.1	9.0
150513	0.1	0.1	1.00E-01	1.00E-07	5	yes	84.2	5.3	10.5
150513	1	0.1	1.00E-01	1.00E-07	5	yes	81.6	9.2	9.2
150513	10	0.1	1.00E-01	1.00E-07	5	yes	78.7	5.1	16.2
150513	0	1	1.00E+00	1.00E-06	5	yes	83.1	6.9	10.0
150513	0.1	1	1.00E+00	1.00E-06	5	yes	80.0	7.0	13.0
150513	1	1	1.00E+00	1.00E-06	5	yes	85.0	5.8	9.2

150513	10	1	1.00E+00	1.00E-06	5	yes	79.7	4.4	15.8
150513	0	10	1.00E+01	1.00E-05	5	yes	0.8	11.3	87.9
150513	0.1	10	1.00E+01	1.00E-05	5	yes	1.3	15.2	83.4
150513	1	10	1.00E+01	1.00E-05	5	yes	1.1	17.6	81.4
150513	10	10	1.00E+01	1.00E-05	5	yes	1.5	16.7	81.9
150520	0	0	0.00E+00	0.00E+00	5	yes	83.8	5.8	10.4
150520	0.1	0	0.00E+00	0.00E+00	5	yes	84.7	3.7	11.6
150520	1	0	0.00E+00	0.00E+00	5	yes	71.9	10.7	17.4
150520	10	0	0.00E+00	0.00E+00	5	yes	63.8	11.8	24.5
150520	0	0.1	1.00E-01	1.00E-07	5	yes	85.4	4.4	10.2
150520	0.1	0.1	1.00E-01	1.00E-07	5	yes	81.2	4.6	14.2
150520	1	0.1	1.00E-01	1.00E-07	5	yes	74.1	8.2	17.7
150520	10	0.1	1.00E-01	1.00E-07	5	yes	69.3	10.0	20.7
150520	0	1	1.00E+00	1.00E-06	5	yes	82.5	4.3	13.2
150520	0.1	1	1.00E+00	1.00E-06	5	yes	71.8	6.3	21.9
150520	1	1	1.00E+00	1.00E-06	5	yes	75.8	5.6	18.6
150520	10	1	1.00E+00	1.00E-06	5	yes	72.8	8.6	18.6
150520	0	10	1.00E+01	1.00E-05	5	yes	1.8	9.0	89.3
150520	0.1	10	1.00E+01	1.00E-05	5	yes	2.3	8.4	89.4
150520	1	10	1.00E+01	1.00E-05	5	yes	1.3	11.8	86.9
150520	10	10	1.00E+01	1.00E-05	5	yes	0.9	12.7	86.4
160603	0	0	0.00E+00	0.00E+00	5	yes	84.5	5.6	10.0
160603	0.1	0	0.00E+00	0.00E+00	5	yes	83.9	5.6	10.6
160603	1	0	0.00E+00	0.00E+00	5	yes	79.0	2.8	18.2
160603	10	0	0.00E+00	0.00E+00	5	yes	78.5	5.8	15.7
160603	0	0.1	1.00E-01	1.00E-07	5	yes	84.3	4.0	11.7
160603	0.1	0.1	1.00E-01	1.00E-07	5	yes	86.5	2.8	10.7
160603	1	0.1	1.00E-01	1.00E-07	5	yes	85.7	1.9	12.3
160603	10	0.1	1.00E-01	1.00E-07	5	yes	83.2	4.7	12.1
160603	0	1	1.00E+00	1.00E-06	5	yes	84.1	2.9	13.0
160603	0.1	1	1.00E+00	1.00E-06	5	yes	83.6	2.9	13.5

160603	1	1	1.00E+00	1.00E-06	5	yes	87.0	1.5	11.5
160603	10	1	1.00E+00	1.00E-06	5	yes	83.8	2.8	13.5
160603	0	10	1.00E+01	1.00E-05	5	yes	0.9	23.0	76.1
160603	0.1	10	1.00E+01	1.00E-05	5	yes	2.8	27.3	69.9
160603	1	10	1.00E+01	1.00E-05	5	yes	1.0	31.4	67.6
160603	10	10	1.00E+01	1.00E-05	5	yes	0.7	34.1	65.2
150520	100	0	0.00E+00	0.00E+00	5	yes	47.6	13.1	39.3
160603	100	0	0.00E+00	0.00E+00	5	yes	42.6	9.96	47.5
171018	0	0	0.00E+00	0.00E+00	5	yes	61.6	8.4	30
171018	100	0	0.00E+00	0.00E+00	5	yes	39.1	6.6	54.3
171018	100	0.1	1.00E-01	1.00E-07	5	yes	40.2	5.7	54.1
171018	100	1	1.00E+00	1.00E-06	5	yes	34.8	5.4	59.8
171018	100	10	1.00E+01	1.00E-05	5	yes	0.7	2.9	96.4
171018	0	0	0.00E+00	0.00E+00	5	yes	79	5.3	15.7
171018	100	0	0.00E+00	0.00E+00	5	yes	45.6	8.4	46.1
171018	100	0.1	1.00E-01	1.00E-07	5	yes	51.7	9.4	38.8
171018	100	1	1.00E+00	1.00E-06	5	yes	46.1	10.5	43.4
171018	100	10	1.00E+01	1.00E-05	5	yes	0.9	2.8	96.3
171018	0	0	0.00E+00	0.00E+00	5	yes	74.5	6.1	19.5
171018	100	0	0.00E+00	0.00E+00	5	yes	45.7	6.9	47.5
171018	100	0.1	1.00E-01	1.00E-07	5	yes	42.3	6	51.6
171018	100	1	1.00E+00	1.00E-06	5	yes	41	7.8	51.2
171018	100	10	1.00E+01	1.00E-05	5	yes	0.7	2.8	96.4

Cell cycle (treatment 3d)

Date	Endoxifen uM	Endoxifen uM	Endoxifen M	Iscador ug/ml	Estradiol	G0/G1	S	G2/M
150618	0	0.00E+00	0.00E+00	0	no	71.3	20.37	7.32
150618	1	1.00E+00	1.00E-06	0	no	79.35	15.36	4.32
150618	1	1.00E+00	1.00E-06	10	no	79.93	12.88	6.95
150618	1	1.00E+00	1.00E-06	100	no	69.09	18.22	9.35
150618	0	0.00E+00	0.00E+00	10	no	67.72	21.68	9.25
150618	0	0.00E+00	0.00E+00	100	no	65.16	22.67	8.54
150618	0	0.00E+00	0.00E+00	0	yes	64.02	29.82	6.13
150618	1	1.00E+00	1.00E-06	0	yes	64.67	30.54	5.47
150618	1	1.00E+00	1.00E-06	10	yes	65.25	25.55	8.08
150618	1	1.00E+00	1.00E-06	100	yes	60.92	27.22	9.96
150618	0	0.00E+00	0.00E+00	10	yes	63.26	27.05	9.72
150618	0	0.00E+00	0.00E+00	100	yes	59.61	27.12	9.73
150625	0	0.00E+00	0.00E+00	0	no	70.35	20.9	9.3
150625	1	1.00E+00	1.00E-06	0	no	79.64	12.96	7.55
150625	1	1.00E+00	1.00E-06	10	no	82.21	14.03	6.37
150625	1	1.00E+00	1.00E-06	100	no	68.91	19.91	8.05
150625	0	0.00E+00	0.00E+00	10	no	71.21	20.7	9.78
150625	0	0.00E+00	0.00E+00	100	no	66.17	21.65	9.51
150625	0	0.00E+00	0.00E+00	0	yes	64.46	29.23	6.53
150625	1	1.00E+00	1.00E-06	0	yes	64.92	27.51	8.02
150625	1	1.00E+00	1.00E-06	10	yes	67.06	24.61	10.51
150625	1	1.00E+00	1.00E-06	100	yes	64.01	25.02	8.9
150625	0	0.00E+00	0.00E+00	10	yes	64.26	26.8	9.63
150625	0	0.00E+00	0.00E+00	100	yes	62.1	26.08	8.92
150629	0	0.00E+00	0.00E+00	0	no	68.1	21.99	9.34
150629	1	1.00E+00	1.00E-06	0	no	80.32	15.39	5.57
150629	1	1.00E+00	1.00E-06	10	no	80.57	12.73	7.44
150629	1	1.00E+00	1.00E-06	100	no	69.33	18.14	9.04

150629	0	0.00E+00	0.00E+00	10	no	69.92	21.4	11.82
150629	0	0.00E+00	0.00E+00	100	no	70.8	20.13	13.78
150629	0	0.00E+00	0.00E+00	0	yes	58.47	29.9	11.12
150629	1	1.00E+00	1.00E-06	0	yes	58.82	30.57	10
150629	1	1.00E+00	1.00E-06	10	yes	62.57	26.88	10.76
150629	1	1.00E+00	1.00E-06	100	yes	61.89	24.19	11.31
150629	0	0.00E+00	0.00E+00	10	yes	61.17	28.14	10.22
150629	0	0.00E+00	0.00E+00	100	yes	61.27	24.18	11.33
150702	0	0.00E+00	0.00E+00	0	no	72.97	19.64	9.08
150702	1	1.00E+00	1.00E-06	0	no	80.45	13.38	7.09
150702	1	1.00E+00	1.00E-06	10	no	81.77	11.91	7.32
150702	1	1.00E+00	1.00E-06	100	no	74.49	15.27	7.75
150702	0	0.00E+00	0.00E+00	10	no	71.12	20.6	8.92
150702	0	0.00E+00	0.00E+00	100	no	68.14	21.49	11.01
150702	0	0.00E+00	0.00E+00	0	yes	65.58	26.89	8.51
150702	1	1.00E+00	1.00E-06	0	yes	65	26.78	8.2
150702	1	1.00E+00	1.00E-06	10	yes	64.82	25.28	9.52
150702	1	1.00E+00	1.00E-06	100	yes	65.19	24.61	8.18
150702	0	0.00E+00	0.00E+00	10	yes	64.18	25.48	10.05
150702	0	0.00E+00	0.00E+00	100	yes	63.15	25.61	8.54
160229	0	0.00E+00	0.00E+00	0	no	81.11	10.58	7.81
160229	1	1.00E+00	1.00E-06	0	no	90.14	4.41	4.07
160229	1	1.00E+00	1.00E-06	10	no	91.19	1.95	5.29
160229	1	1.00E+00	1.00E-06	100	no	82.42	7.57	6.8
160229	0	0.00E+00	0.00E+00	10	no	82.41	9.79	7.21
160229	0	0.00E+00	0.00E+00	100	no	80.89	9.64	10.66
160229	0	0.00E+00	0.00E+00	0	yes	74.28	18.01	11.36
160229	1	1.00E+00	1.00E-06	0	yes	71.72	15.52	12.12
160229	1	1.00E+00	1.00E-06	10	yes	72.58	15	11.81
160229	1	1.00E+00	1.00E-06	100	yes	73.41	15.29	9.52
160229	0	0.00E+00	0.00E+00	10	yes	72.81	15.87	11.19

160229	0	0.00E+00	0.00E+00	100	yes	71.84	15.54	11.77
160321	0	0.00E+00	0.00E+00	0	no	77.6	12.47	7.58
160321	1	1.00E+00	1.00E-06	0	no	87.41	5.56	3.5
160321	1	1.00E+00	1.00E-06	10	no	87.45	3.77	6.44
160321	1	1.00E+00	1.00E-06	100	no	80.46	10.05	6.27
160321	0	0.00E+00	0.00E+00	10	no	77.72	11.7	8.65
160321	0	0.00E+00	0.00E+00	100	no	74.81	11.69	11.21
160321	0	0.00E+00	0.00E+00	0	yes	67.35	21.72	8.38
160321	1	1.00E+00	1.00E-06	0	yes	67.3	20.82	9.35
160321	1	1.00E+00	1.00E-06	10	yes	71.08	15.63	11.61
160321	1	1.00E+00	1.00E-06	100	yes	70.34	16.35	10.05
160321	0	0.00E+00	0.00E+00	10	yes	69.98	16.14	12.54
160321	0	0.00E+00	0.00E+00	100	yes	68.35	18.61	10.62
160418	0	0.00E+00	0.00E+00	0	no	81.84	7.61	9.18
160418	1	1.00E+00	1.00E-06	0	no	88	4.07	6.45
160418	1	1.00E+00	1.00E-06	10	no	87.49	5.41	5.44
160418	1	1.00E+00	1.00E-06	100	no	78.29	10.4	9.2
160418	0	0.00E+00	0.00E+00	10	no	80.26	9.06	9.45
160418	0	0.00E+00	0.00E+00	100	no	74.26	10.97	12.66
160418	0	0.00E+00	0.00E+00	0	yes	71.39	15.45	11.63
160418	1	1.00E+00	1.00E-06	0	yes	71.56	15.21	12.69
160418	1	1.00E+00	1.00E-06	10	yes	73.2	13.81	12.35
160418	1	1.00E+00	1.00E-06	100	yes	69.3	15.17	12.98
160418	0	0.00E+00	0.00E+00	10	yes	71.6	14.08	13.5
160418	0	0.00E+00	0.00E+00	100	yes	70.19	15.02	13.53
