# Supplementary material for: Absence of herb-drug interactions of mistletoe with the tamoxifen metabolite (E/Z)-endoxifen and cytochrome P450 3A4/5 and 2D6 in vitro
Source: BMC Complement Altern Med. 2019 Jan 18;19:23. doi: 10.1186/s12906-019-2439-2 (PMC6339413; doi:10.1186/s12906-019-2439-2)
Supplement: Supplementary file 3 — Data_CYP2D6_and_CYP3A4-5_inhibition assays. (PDF 105 kb) [file 12906_2019_2439_MOESM3_ESM.pdf]

Data Fig 6A_CYP2D6 inhibition assays_Endoxifen_Metabolite_Mean of 3 assays_SD_SE

**Assay I**			**Assay II**		
Endoxifen (metabolite)			Endoxifen (metabolite)		
	Peak Area Ratio	% metabolite vs NC		Peak Area Ratio	% metabolite vs NC
D_Test Iscador P 10 mg_500 µg/mL	3.60616E-05	89.1	D_Test Iscador P 10 mg_500 µg/mL	7.21398E-06	43.8
D_Test Iscador P 10 mg_100 µg/mL	4.13965E-05	102.3	D_Test Iscador P 10 mg_100 µg/mL	1.95476E-05	118.6
D_Test Iscador P 10 mg_10 µg/mL	3.78969E-05	93.6	D_Test Iscador P 10 mg_10 µg/mL	1.96541E-05	119.3
D_Test Iscador P 10 mg_5µg/mL	3.54045E-05	87.5	D_Test Iscador P 10 mg_5µg/mL	2.17025E-05	131.7
D_Test Iscador P 10 mg_1 µg/mL	4.22246E-05	104.3	D_Test Iscador P 10 mg_1 µg/mL	1.6304E-05	98.9
D_Test Iscador P 10 mg_0.1 µg/mL	3.68969E-05	91.2	D_Test Iscador P 10 mg_0.1 µg/mL	2.15621E-05	130.8
	Peak Area Ratio	metabolite vs NC		Peak Area Ratio	; metabolite vs N(
D_Test Iscador Qu 5 mg_500 µg/mL	4.25015E-05	105.0	D_Test Iscador Qu 5 mg_500 µg/mL	1.72586E-05	104.7
D_Test Iscador Qu 5 mg_100 µg/mL	5.1676E-05	127.7	D_Test Iscador Qu 5 mg_100 µg/mL	1.83713E-05	111.5
D_Test Iscador Qu 5 mg_10 µg/mL	3.53096E-05	87.2	D_Test Iscador Qu 5 mg_10 µg/mL	1.98354E-05	120.4
D_Test Iscador Qu 5 mg_5 µg/mL	4.17018E-05	103.0	D_Test Iscador Qu 5 mg_5 µg/mL	1.81764E-05	110.3
D_Test Iscador Qu 5 mg_1 µg/mL	4.16317E-05	102.8	D_Test Iscador Qu 5 mg_1 µg/mL	2.45853E-05	149.2
D_Test Iscador Qu 5 mg_0.1 µg/mL	4.21081E-05	104.0	D_Test Iscador Qu 5 mg_0.1 µg/mL	1.89268E-05	114.9
	Peak Area Ratio	metabolite vs NC		Peak Area Ratio	; metabolite vs N(
D_Test Iscador M 5 mg_500 µg/mL	3.91586E-05	96.7	D_Test Iscador M 5 mg_500 µg/mL	1.63952E-05	99.5
D_Test Iscador M 5 mg_100 µg/mL	3.74561E-05	92.5	D_Test Iscador M 5 mg_100 µg/mL	2.07329E-05	125.8
D_Test Iscador M 5 mg_10 µg/mL	3.95348E-05	97.7	D_Test Iscador M 5 mg_10 µg/mL	1.65889E-05	100.7
D_Test Iscador M 5 mg_5 µg/mL	4.38595E-05	108.4	D_Test Iscador M 5 mg_5 µg/mL	1.72681E-05	104.8
D_Test Iscador M 5 mg_1 µg/mL	3.88095E-05	95.9	D_Test Iscador M 5 mg_1 µg/mL	1.50332E-05	91.2
D_Test Iscador M 5 mg_0.1 µg/mL	4.29956E-05	106.2	D_Test Iscador M 5 mg_0.1 µg/mL	2.04076E-05	123.8
	Peak Area Ratio	metabolite vs NC		Peak Area Ratio	; metabolite vs N(
D_Negative Control 1-1_no inhibitors	4.4456E-05	109.8	D_Negative Control 1-1_no inhibitors	1.6213E-05	98.4
D_Negative Control 1-2_no inhibitors	4.05463E-05	100.2	D_Negative Control 1-2_no inhibitors	1.60233E-05	97.2
D_Negative Control 1-3_no inhibitors	3.64346E-05	90.0	D_Negative Control 1-3_no inhibitors	1.7201E-05	104.4
D_Negative Control 2_no inhibitors/no microsomes	0.00	0.00	ative Control 2_no inhibitors/no micr(	0.00	0.00
)_Negative Control 3_no inhibitors/no microsomes/no NADPI	0.00	0.00	:ontrol 3_no inhibitors/no microsome	0.00	0.00
Average Negative controls	4.0479E-05		Average Negative controls	1.64791E-05	100

U. Weissenstein, M. Kunz, M. Oufir, J.T. Wang, M. Hamburger, K. Urech, U. Regueiro, S, Baumgartner

Assay III			Mean	SD	SE
Endoxifen (metabolite)					
	Peak Area Ratio	% metabolite			
D_Test Iscador P 10 mg_500 µg/mL	3.43457E-05	77.2	**70.0**	**23.5**	13.56
D_Test Iscador P 10 mg_100 µg/mL	4.3534E-05	97.9	**106.2**	**10.9**	6.32
D_Test Iscador P 10 mg_10 µg/mL	4.39669E-05	98.8	**103.9**	**13.6**	7.83
D_Test Iscador P 10 mg_5µg/mL	6.68009E-05	150.2	**123.1**	**32.2**	18.60
D_Test Iscador P 10 mg_1 µg/mL	5.69661E-05	128.0	**110.4**	**15.5**	8.94
D_Test Iscador P 10 mg_0.1 µg/mL	4.5065E-05	101.3	**107.8**	**20.6**	11.91
C	Peak Area Ratio	netabolite vs NC			
D_Test Iscador Qu 5 mg_500 µg/mL	4.31629E-05	97.0	**102.2**	**4.53**	2.62
D_Test Iscador Qu 5 mg_100 µg/mL	4.22026E-05	94.9	**111.3**	**16.4**	9.47
D_Test Iscador Qu 5 mg_10 µg/mL	4.77204E-05	107.3	**105.0**	**16.7**	9.64
D_Test Iscador Qu 5 mg_5 µg/mL	5.78444E-05	130.0	**114.4**	**14.0**	8.06
D_Test Iscador Qu 5 mg_1 µg/mL	4.89721E-05	110.1	**120.7**	**24.9**	14.39
D_Test Iscador Qu 5 mg_0.1 µg/mL	5.33684E-05	120.0	**112.9**	**8.14**	4.70
C	Peak Area Ratio	netabolite vs NC			
D_Test Iscador M 5 mg_500 µg/mL	4.31235E-05	96.9	**97.7**	**1.54**	0.89
D_Test Iscador M 5 mg_100 µg/mL	4.88613E-05	109.8	**109.4**	**16.6**	9.61
D_Test Iscador M 5 mg_10 µg/mL	4.32642E-05	97.2	**98.5**	**1.86**	1.08
D_Test Iscador M 5 mg_5 µg/mL	4.40275E-05	99.0	**104.0**	**4.74**	2.74
D_Test Iscador M 5 mg_1 µg/mL	4.14447E-05	93.2	**93.4**	**2.34**	1.35
D_Test Iscador M 5 mg_0.1 µg/mL	4.46695E-05	100.4	**110.2**	**12.2**	7.05
C	Peak Area Ratio	netabolite vs NC			
)_Negative Control 1-1_no inhibitor	4.64481E-05	104.4	**104.2**	**5.72**	3.30
)_Negative Control 1-2_no inhibitor	4.40081E-05	98.9	**98.8**	**1.47**	0.85
)_Negative Control 1-3_no inhibitor	4.30115E-05	96.7	**97.0**	**7.19**	4.15
tive Control 2_no inhibitors/no micr	1.86875E-05	42.0	**14.00**	**24.3**	14.00
ontrol 3_no inhibitors/no microsom	2.24818E-05	50.5	**16.84**	**29.2**	16.84
Average Negative controls	4.44892E-05	100	**100.0**	**0.0**	0.000

Data Fig 6B_CYP3A4-5 inhibition assays_Endoxifen_Metabolite_Mean of 3 assays_SD_SE

Assay I			Assay II		
**Endoxifen (metabolite)**			**Endoxifen (metabolite)**		
	**Peak Area Ratio**	**% metabolite vs NC**		**Peak Area Ratio**	**% metabolite vs NC**
TE_Test Iscador P 10 mg_500 µg/mL	3.57113E-05	86.5	TE_Test Iscador P 10 mg_500 µg/mL	3.23578E-05	81.0
TE_Test Iscador P 10 mg_100 µg/mL	3.88185E-05	94.0	TE_Test Iscador P 10 mg_100 µg/mL	5.11774E-05	128.2
TE_Test Iscador P 10 mg_10 µg/mL	4.17151E-05	101.0	TE_Test Iscador P 10 mg_10 µg/mL	4.85964E-05	121.7
TE_Test Iscador P 10 mg_5µg/mL	4.87448E-05	118.0	TE_Test Iscador P 10 mg_5µg/mL	4.59791E-05	115.1
TE_Test Iscador P 10 mg_1 µg/mL	3.89346E-05	94.3	TE_Test Iscador P 10 mg_1 µg/mL	4.74794E-05	118.9
TE_Test Iscador P 10 mg_0.1 µg/mL	4.90767E-05	118.8	TE_Test Iscador P 10 mg_0.1 µg/mL	5.41175E-05	135.5
	**Peak Area Ratio**	**% metabolite vs NC**		**Peak Area Ratio**	**% metabolite vs NC**
TE_Test Iscador Qu 5 mg_500 µg/mL	-	-	TE_Test Iscador Qu 5 mg_500 µg/mL	4.97212E-05	124.5
TE_Test Iscador Qu 5 mg_100 µg/mL	4.10534E-05	99.4	TE_Test Iscador Qu 5 mg_100 µg/mL	5.1219E-05	128.3
TE_Test Iscador Qu 5 mg_10 µg/mL	3.93644E-05	95.3	TE_Test Iscador Qu 5 mg_10 µg/mL	5.27174E-05	132.0
TE_Test Iscador Qu 5 mg_5 µg/mL	4.49849E-05	108.9	TE_Test Iscador Qu 5 mg_5 µg/mL	4.35692E-05	109.1
TE_Test Iscador Qu 5 mg_1 µg/mL	3.72907E-05	90.3	TE_Test Iscador Qu 5 mg_1 µg/mL	4.93094E-05	123.5
TE_Test Iscador Qu 5 mg_0.1 µg/mL	4.29758E-05	104.1	TE_Test Iscador Qu 5 mg_0.1 µg/mL	4.38854E-05	109.9
	**Peak Area Ratio**	**% metabolite vs NC**		**Peak Area Ratio**	**% metabolite vs NC**
TE_Test Iscador M 5 mg_500 µg/mL	3.63107E-05	87.9	TE_Test Iscador M 5 mg_500 µg/mL	3.58424E-05	89.8
TE_Test Iscador M 5 mg_100 µg/mL	5.08802E-05	123.2	TE_Test Iscador M 5 mg_100 µg/mL	4.80585E-05	120.3
TE_Test Iscador M 5 mg_10 µg/mL	4.07728E-05	98.7	TE_Test Iscador M 5 mg_10 µg/mL	-	-
TE_Test Iscador M 5 mg_5 µg/mL	4.42298E-05	107.1	TE_Test Iscador M 5 mg_5 µg/mL	4.17972E-05	104.7
TE_Test Iscador M 5 mg_1 µg/mL	-	-	TE_Test Iscador M 5 mg_1 µg/mL	-	107.7
TE_Test Iscador M 5 mg_0.1 µg/mL	3.65532E-05	88.5	TE_Test Iscador M 5 mg_0.1 µg/mL	-	-
	**Peak Area Ratio**	**% metabolite vs NC**		**Peak Area Ratio**	**% metabolite vs NC**
TE_Negative Control 1-1_no inhibitors	4.21314E-05	102.0	TE_Negative Control 1-1_no inhibitors	3.92572E-05	98.3
TE_Negative Control 1-2_no inhibitors	4.17027E-05	101.0	TE_Negative Control 1-2_no inhibitors	3.73476E-05	93.5
TE_Negative Control 1-3_no inhibitors	4.00693E-05	97.0	TE_Negative Control 1-3_no inhibitors	4.31965E-05	108.2
TE_Negative Control 2_no inhibitors/no microsomes	0.00	0.00	Negative Control 2_no inhibitors/no microsor	0.00	0.00
Negative Control 3_no inhibitors/no microsomes/no NAI	0.00	0.00	ive Control 3_no inhibitors/no microsomes/n	0.00	0.00
Average Negative controls	0.00004130	100	Average Negative controls	0.00003993	100.0

Assay III			Mean	SD	SE
Endoxifen (metabolite)					
	Peak Area Ratio	% metabolite vs NC			
TE_Test Iscador P 10 mg_500 µg/mL	3.71197E-05	85.0	**84.2**	**2.81**	1.62
TE_Test Iscador P 10 mg_100 µg/mL	4.35216E-05	99.7	**107.3**	**18.3**	10.57
TE_Test Iscador P 10 mg_10 µg/mL	4.46759E-05	102.3	**108.3**	**11.6**	6.69
TE_Test Iscador P 10 mg_5µg/mL	4.48841E-05	102.8	**112.0**	**8.1**	4.67
TE_Test Iscador P 10 mg_1 µg/mL	5.41371E-05	124.0	**112.4**	**15.9**	9.17
TE_Test Iscador P 10 mg_0.1 µg/mL	4.81502E-05	110.3	**121.5**	**12.8**	7.42
	Peak Area Ratio	% metabolite vs NC			
TE_Test Iscador Qu 5 mg_500 µg/mL	5.39596E-05	123.6	**124.0**	**0.7**	0.47
TE_Test Iscador Qu 5 mg_100 µg/mL	4.9582E-05	113.5	**113.7**	**14.4**	8.33
TE_Test Iscador Qu 5 mg_10 µg/mL	5.04966E-05	115.6	**114.3**	**18.4**	10.62
TE_Test Iscador Qu 5 mg_5 µg/mL	5.41894E-05	124.1	**114.0**	**8.7**	5.03
TE_Test Iscador Qu 5 mg_1 µg/mL	5.85422E-05	134.1	**115.9**	**22.8**	13.18
TE_Test Iscador Qu 5 mg_0.1 µg/mL	4.97191E-05	113.9	**109.3**	**4.9**	2.85
	Peak Area Ratio	% metabolite vs NC			
TE_Test Iscador M 5 mg_500 µg/mL	4.28273E-05	98.1	**91.9**	**5.4**	3.12
TE_Test Iscador M 5 mg_100 µg/mL	5.0164E-05	114.9	**119**	**4.2**	2.44
TE_Test Iscador M 5 mg_10 µg/mL	4.28707E-05	98.2	**98.4**	**0.4**	0.27
TE_Test Iscador M 5 mg_5 µg/mL	-	-	**106**	**1.7**	1.21
TE_Test Iscador M 5 mg_1 µg/mL	5.05595E-05	115.8	**112**	**5.7**	4.04
TE_Test Iscador M 5 mg_0.1 µg/mL	4.45102E-05	101.9	**95.2**	**9.5**	6.71
	Peak Area Ratio	% metabolite vs NC			
TE_Negative Control 1-1_no inhibitors	4.35539E-05	99.7	**100.0**	**1.87**	1.08
TE_Negative Control 1-2_no inhibitors	4.43937E-05	101.7	**98.7**	**4.51**	2.60
TE_Negative Control 1-3_no inhibitors	4.3061E-05	98.6	**101.3**	**6.03**	3.48
Negative Control 2_no inhibitors/no microsoı	0.00	0.00	**0.00**	**0.00**	0.00
ive Control 3_no inhibitors/no microsomes/r	0.00	0.00	**0.00**	**0.00**	0.00
Average Negative controls	4.36695E-05	100	**100**	**0.00**	0.00

Data Fig S1_CYP2D6 inhibition assays_Dextrorphan_Metabolite_Mean of 3 assays_SD_SE

**Assay I**			**Assay II**		
Dextrorphan (metabolite)			Dextrorphan (metabolite)		
	Peak Area Ratio	% metabolite vs NC		Peak Area Ratio	% metabolite vs NC
D_Test Iscador P 10 mg_500 µg/mL	0.014208097	94.4	D_Test Iscador P 10 mg_500 µg/mL	0.011565397	67.5
D_Test Iscador P 10 mg_100 µg/mL	0.015814367	105.1	D_Test Iscador P 10 mg_100 µg/mL	0.016361201	95.6
D_Test Iscador P 10 mg_10 µg/mL	0.015028411	99.8	D_Test Iscador P 10 mg_10 µg/mL	0.01626224	95.0
D_Test Iscador P 10 mg_5µg/mL	0.01486649	98.8	D_Test Iscador P 10 mg_5µg/mL	0.017125527	100.0
D_Test Iscador P 10 mg_1 µg/mL	0.015336473	101.9	D_Test Iscador P 10 mg_1 µg/mL	0.017291943	101.0
D_Test Iscador P 10 mg_0.1 µg/mL	0.014776322	98.2	D_Test Iscador P 10 mg_0.1 µg/mL	0.015527767	90.7
	Peak Area Ratio	% metabolite vs NC		Peak Area Ratio	% metabolite vs NC
D_Test Iscador Qu 5 mg_500 µg/mL	0.013341404	88.6	D_Test Iscador Qu 5 mg_500 µg/mL	0.012937646	75.6
D_Test Iscador Qu 5 mg_100 µg/mL	0.013205066	87.7	D_Test Iscador Qu 5 mg_100 µg/mL	0.014724655	86.0
D_Test Iscador Qu 5 mg_10 µg/mL	0.015431764	102.5	D_Test Iscador Qu 5 mg_10 µg/mL	0.013770358	80.4
D_Test Iscador Qu 5 mg_5 µg/mL	0.013335287	88.6	D_Test Iscador Qu 5 mg_5 µg/mL	0.016662382	97.3
D_Test Iscador Qu 5 mg_1 µg/mL	0.012561886	83.5	D_Test Iscador Qu 5 mg_1 µg/mL	0.015424235	90.1
D_Test Iscador Qu 5 mg_0.1 µg/mL	0.013288851	88.3	D_Test Iscador Qu 5 mg_0.1 µg/mL	0.016091145	94.0
	Peak Area Ratio	% metabolite vs NC		Peak Area Ratio	% metabolite vs NC
D_Test Iscador M 5 mg_500 µg/mL	0.011586939	77.0	D_Test Iscador M 5 mg_500 µg/mL	0.013112596	76.6
D_Test Iscador M 5 mg_100 µg/mL	0.013301942	88.4	D_Test Iscador M 5 mg_100 µg/mL	0.013860267	80.9
D_Test Iscador M 5 mg_10 µg/mL	0.014062342	93.4	D_Test Iscador M 5 mg_10 µg/mL	0.01305638	76.3
D_Test Iscador M 5 mg_5 µg/mL	0.014895997	99.0	D_Test Iscador M 5 mg_5 µg/mL	0.014397467	84.1
D_Test Iscador M 5 mg_1 µg/mL	0.013970371	92.8	D_Test Iscador M 5 mg_1 µg/mL	0.013754865	80.3
D_Test Iscador M 5 mg_0.1 µg/mL	0.014813049	98.4	D_Test Iscador M 5 mg_0.1 µg/mL	0.0139601	81.5
		% metabolite vs NC			% metabolite vs NC
D_Negative Control 1-1_no inhibitors	0.015269822	101.4	D_Negative Control 1-1_no inhibitors	0.016992498	99.2
D_Negative Control 1-2_no inhibitors	0.015064957	100.1	D_Negative Control 1-2_no inhibitors	0.016852928	98.4
D_Negative Control 1-3_no inhibitors	0.014820281	98.5	D_Negative Control 1-3_no inhibitors	0.01752154	102.3
egative Control 2_no inhibitors/no microso	7.94889E-05	0.53	ative Control 2_no inhibitors/no micr	7.84442E-05	0.46
e Control 3_no inhibitors/no microsomes/	6.36886E-05	0.42	Control 3_no inhibitors/no microsome	4.97233E-05	0.29
		% metabolite vs NC			% metabolite vs NC
Average Negative controls	0.015051687		Average Negative controls	0.017122322	100

Assay III			Mean	SD	SE
Dextrorphan (metabolite)					
	Peak Area Ratio	% metabolite vs NC			
D_Test Iscador P 10 mg_500 µg/mL	0.141899671	91.7	**84.5**	**14.8**	8.53
D_Test Iscador P 10 mg_100 µg/mL	0.152889254	98.8	**99.8**	**4.84**	2.79
D_Test Iscador P 10 mg_10 µg/mL	0.157569351	101.8	**98.9**	**3.52**	2.03
D_Test Iscador P 10 mg_5µg/mL	0.157739425	101.9	**100.2**	**1.59**	0.92
D_Test Iscador P 10 mg_1 µg/mL	0.15607049	100.8	**101.2**	**0.57**	0.33
D_Test Iscador P 10 mg_0.1 µg/mL	0.163160198	105.4	**98.1**	**7.37**	4.25
	Peak Area Ratio	% metabolite vs NC			
D_Test Iscador Qu 5 mg_500 µg/mL	0.152076673	98.3	**87.5**	**11.39**	6.58
D_Test Iscador Qu 5 mg_100 µg/mL	0.157943709	102.1	**91.9**	**8.81**	5.09
D_Test Iscador Qu 5 mg_10 µg/mL	0.163418313	105.6	**96.2**	**13.73**	7.93
D_Test Iscador Qu 5 mg_5 µg/mL	0.157926864	102.0	**96.0**	**6.82**	3.94
D_Test Iscador Qu 5 mg_1 µg/mL	0.14513121	93.8	**89.1**	**5.23**	3.02
D_Test Iscador Qu 5 mg_0.1 µg/mL	0.156292574	101.0	**94.4**	**6.36**	3.67
	Peak Area Ratio	% metabolite vs NC			
D_Test Iscador M 5 mg_500 µg/mL	0.148206864	95.8	**83.1**	**10.96**	6.33
D_Test Iscador M 5 mg_100 µg/mL	0.149782759	96.8	**88.7**	**7.92**	4.57
D_Test Iscador M 5 mg_10 µg/mL	0.155753751	100.6	**90.1**	**12.53**	7.23
D_Test Iscador M 5 mg_5 µg/mL	0.147654642	95.4	**92.8**	**7.77**	4.49
D_Test Iscador M 5 mg_1 µg/mL	0.150028031	96.9	**90.0**	**8.65**	4.99
D_Test Iscador M 5 mg_0.1 µg/mL	0.15322061	99.0	**93.0**	**9.92**	5.73
		% metabolite vs NC			
)_Negative Control 1-1_no inhibitor	0.151833594	98.1	**99.6**	**1.70**	0.98
)_Negative Control 1-2_no inhibitor	0.148491204	95.9	**98.2**	**2.09**	1.20
)_Negative Control 1-3_no inhibitor	0.163975889	106.0	**102.2**	**3.74**	2.16
tive Control 2_no inhibitors/no mici	0.010673354	6.90	**2.63**	**3.70**	2.13
ontrol 3_no inhibitors/no microsom	0.009634672	6.23	**2.31**	**3.39**	1.96
		% metabolite vs NC			
Average Negative controls	0.154766896	100	**100.0**	**0.0**	0.00

Data Fig S2_CYP2D6 inhibition assays by Quinidin_Endoxifen_Metabolite_Mean of 3 assays_SD_SE

Assay I			Assay II		
Endoxifen (metabolite)			Endoxifen (metabolite)		
	Peak Area Ratio	% metabolite vs NC		Peak Area Ratio	% metabolite vs NC
D_Positive Control_Quinidine 100 uM	0.00	0.00	D_Positive Control_Quinidine 100 uM	0.00	0.00
D_Positive Control_Quinidine 10 uM	2.01993E-05	49.9	D_Positive Control_Quinidine 10 uM	6.51387E-06	39.5
D_Positive Control_Quinidine 5 uM	2.20493E-05	54.5	D_Positive Control_Quinidine 5 uM	7.6237E-06	46.3
D_Positive Control_Quinidine 2 uM	2.51806E-05	62.2	D_Positive Control_Quinidine 2 uM	8.07856E-06	49.0
D_Positive Control_Quinidine 1 uM	2.55626E-05	63.2	D_Positive Control_Quinidine 1 uM	9.69827E-06	58.9
D_Positive Control_Quinidine 0.1 uM	2.84574E-05	70.3	D_Positive Control_Quinidine 0.1 uM	1.38156E-05	83.8
D_Negative Control 1-1_no inhibitors	4.4456E-05	109.8	D_Negative Control 1-1_no inhibitors	1.6213E-05	98.4

Assay III			Mean	SD	SE
Endoxifen (metabolite)					
	Peak Area Ratio	% metabolite vs NC			
D_Positive Control_Quinidine 100 uM	7.35921E-06	16.5	**5.51**	**9.55**	5.51
D_Positive Control_Quinidine 10 uM	1.15879E-05	26.0	**38.5**	**12.0**	6.91
D_Positive Control_Quinidine 5 uM	2.17554E-05	48.9	**49.9**	**4.19**	2.42
D_Positive Control_Quinidine 2 uM	2.33783E-05	52.5	**54.6**	**6.83**	3.94
D_Positive Control_Quinidine 1 uM	2.52793E-05	56.8	**59.6**	**3.23**	1.87
D_Positive Control_Quinidine 0.1 uM	3.95828E-05	89.0	**81.0**	**9.64**	5.57
D_Negative Control 1-1_no inhibitors	4.64481E-05	104.4	**104.2**	**5.72**	3.30

Data Fig S3_CYP2D6 inhibition assays by Quinidine_Dextrorphan_Metabolite_Mean of 3 assays_SD_SE

**Assay I**			**Assay II**		
Endoxifen (metabolite)			Endoxifen (metabolite)		
	Peak Area Ratio	% metabolite vs NC		Peak Area Ratio	% metabolite vs NC
D_Positive Control_Quinidine 100 uM	0.000212752	1.41	D_Positive Control_Quinidine 100 uM	0.000274118	1.60
D_Positive Control_Quinidine 10 uM	0.000683177	4.54	D_Positive Control_Quinidine 10 uM	0.000820142	4.79
D_Positive Control_Quinidine 5 uM	0.00114825	7.63	D_Positive Control_Quinidine 5 uM	0.001265521	7.39
D_Positive Control_Quinidine 2 uM	0.002349049	15.6	D_Positive Control_Quinidine 2 uM	0.002366089	13.8
D_Positive Control_Quinidine 1 uM	0.003780586	25.1	D_Positive Control_Quinidine 1 uM	0.004402311	25.7
D_Positive Control_Quinidine 0.1 uM	0.010918579	72.5	D_Positive Control_Quinidine 0.1 uM	0.013487882	78.8
D_Negative Control 1-1_no inhibitors	0.015269822	101.4	D_Negative Control 1-1_no inhibitors	0.016992498	99.2

**Assay III**			**Mean**	**SD**	**SE**
Endoxifen (metabolite)					
	Peak Area Ratio	% metabolite vs NC			
D_Positive Control_Quinidine 100 uM	0.006682118	4.32	**2.44**	**1.63**	0.938
D_Positive Control_Quinidine 10 uM	0.01072527	6.93	**5.42**	**1.31**	0.759
D_Positive Control_Quinidine 5 uM	0.014205437	9.18	**8.07**	**0.97**	0.560
D_Positive Control_Quinidine 2 uM	0.023559882	15.2	**14.9**	**0.94**	0.543
D_Positive Control_Quinidine 1 uM	0.039926759	25.8	**25.5**	**0.37**	0.214
D_Positive Control_Quinidine 0.1 uM	0.12451359	80.5	**77.3**	**4.17**	2.407
D_Negative Control 1-1_no inhibitors	0.151833594	98.1	**99.6**	**1.70**	0.982

Data Fig S4_CYP3A4-5 inhibition assays_6BetaHT_Metabolite_Mean of 3 assays_SD_SE

Assay I			Assay II		
**6BHT (metabolite)**			**6BHT (metabolite)**		
	**Peak Area Ratio**	**% metabolite vs NC**		**Peak Area Ratio**	**% metabolite vs NC**
TE_Test Iscador P 10 mg_500 µg/mL	0.00918256	70.3	TE_Test Iscador P 10 mg_500 µg/mL	0.008947753	36.0
TE_Test Iscador P 10 mg_100 µg/mL	0.011075095	84.8	TE_Test Iscador P 10 mg_100 µg/mL	0.018450672	74.3
TE_Test Iscador P 10 mg_10 µg/mL	0.018078525	138.4	TE_Test Iscador P 10 mg_10 µg/mL	-	-
TE_Test Iscador P 10 mg_5µg/mL	0.019729599	151.0	TE_Test Iscador P 10 mg_5µg/mL	0.011266651	45.4
TE_Test Iscador P 10 mg_1 µg/mL	-	-	TE_Test Iscador P 10 mg_1 µg/mL	0.021306142	85.8
TE_Test Iscador P 10 mg_0.1 µg/mL	-	-	TE_Test Iscador P 10 mg_0.1 µg/mL	0.02855527	114.9
	**Peak Area Ratio**	**% metabolite vs NC**		**Peak Area Ratio**	**% metabolite vs NC**
TE_Test Iscador Qu 5 mg_500 µg/mL	0.00320392	24.5	TE_Test Iscador Qu 5 mg_500 µg/mL	0.006491894	26.1
TE_Test Iscador Qu 5 mg_100 µg/mL	0.006875019	52.6	TE_Test Iscador Qu 5 mg_100 µg/mL	0.007022276	28.3
TE_Test Iscador Qu 5 mg_10 µg/mL	0.01305385	99.9	TE_Test Iscador Qu 5 mg_10 µg/mL	-	-
TE_Test Iscador Qu 5 mg_5 µg/mL	-	-	TE_Test Iscador Qu 5 mg_5 µg/mL	0.02154394	86.7
TE_Test Iscador Qu 5 mg_1 µg/mL	0.013564209	103.8	TE_Test Iscador Qu 5 mg_1 µg/mL	0.016468292	66.3
TE_Test Iscador Qu 5 mg_0.1 µg/mL	-	-	TE_Test Iscador Qu 5 mg_0.1 µg/mL	0.018371759	74.0
	**Peak Area Ratio**	**% metabolite vs NC**		**Peak Area Ratio**	**% metabolite vs NC**
TE_Test Iscador M 5 mg_500 µg/mL	0.007739921	59.2	TE_Test Iscador M 5 mg_500 µg/mL	0.009047538	36.4
TE_Test Iscador M 5 mg_100 µg/mL	0.006545399	50.1	TE_Test Iscador M 5 mg_100 µg/mL	0.010206931	41.1
TE_Test Iscador M 5 mg_10 µg/mL	-	-	TE_Test Iscador M 5 mg_10 µg/mL	-	-
TE_Test Iscador M 5 mg_5 µg/mL	0.007768031	59.5	TE_Test Iscador M 5 mg_5 µg/mL	-	-
TE_Test Iscador M 5 mg_1 µg/mL	0.013688362	104.8	TE_Test Iscador M 5 mg_1 µg/mL	0.021213463	85.4
TE_Test Iscador M 5 mg_0.1 µg/mL	0.013350574	102.2	TE_Test Iscador M 5 mg_0.1 µg/mL	-	-
	**Peak Area Ratio**	**% metabolite vs NC**		**Peak Area Ratio**	**% metabolite vs NC**
TE_Negative Control 1-1_no inhibitors	0.012655482	96.9	TE_Negative Control 1-1_no inhibitors	-	-
TE_Negative Control 1-2_no inhibitors	-	-	TE_Negative Control 1-2_no inhibitors	-	-
TE_Negative Control 1-3_no inhibitors	0.013476017	103.1	TE_Negative Control 1-3_no inhibitors	0.02484259	-
E_Negative Control 2_no inhibitors/no microsome	0.00	0.00	gative Control 2_no inhibitors/no micrc	0.00	0.00
gative Control 3_no inhibitors/no microsomes/no N	0.00	0.00	Control 3_no inhibitors/no microsome	0.00	0.00
	**Peak Area Ratio**	**% metabolite vs NC**		**Peak Area Ratio**	**% metabolite vs NC**
		0			
Average Negative controls	0.01306575	100	Average Negative controls	0.02484259	100.0

U. Weissenstein, M. Kunz, M. Oufir, J.T. Wang, M. Hamburger, K. Urech, U. Regueiro, S, Baumgartner

Assay III			Mean	SD	SE
6BHT (metabolite)					
	Peak Area Ratio	% metabolite vs NC			
TE_Test Iscador P 10 mg_500 µg/mL	0.054987651	55.5	**53.9**	**17.19**	9.92
TE_Test Iscador P 10 mg_100 µg/mL	-	-	**79.5**	**7.42**	5.25
TE_Test Iscador P 10 mg_10 µg/mL	0.060730912	61.3	**100**	**54.48**	38.5
TE_Test Iscador P 10 mg_5µg/mL	0.098856935	99.8	**98.7**	**52.83**	30.5
TE_Test Iscador P 10 mg_1 µg/mL	-	-	**85.8**	-	-
TE_Test Iscador P 10 mg_0.1 µg/mL	0.104865961	105.9	**110**	**6.41**	4.53
	Peak Area Ratio	% metabolite vs NC			
TE_Test Iscador Qu 5 mg_500 µg/mL	0.034597475	34.9	**28.5**	**5.60**	3.23
TE_Test Iscador Qu 5 mg_100 µg/mL	0.047440611	47.9	**42.9**	**12.91**	7.46
TE_Test Iscador Qu 5 mg_10 µg/mL	0.068221993	68.9	**84.4**	**21.94**	15.5
TE_Test Iscador Qu 5 mg_5 µg/mL	0.073969412	74.7	**80.7**	**8.51**	6.02
TE_Test Iscador Qu 5 mg_1 µg/mL	0.097044188	98.0	**89.4**	**20.19**	11.7
TE_Test Iscador Qu 5 mg_0.1 µg/mL	-	-	**74.0**	-	-
	Peak Area Ratio	% metabolite vs NC			
TE_Test Iscador M 5 mg_500 µg/mL	0.057689422	58.2	**51.3**	**12.9**	7.45
TE_Test Iscador M 5 mg_100 µg/mL	0.052339284	52.8	**48.0**	**6.2**	3.55
TE_Test Iscador M 5 mg_10 µg/mL	0.058158691	58.7	**58.7**	-	-
TE_Test Iscador M 5 mg_5 µg/mL	-	-	**59.5**	-	-
TE_Test Iscador M 5 mg_1 µg/mL	-	-	**95.1**	**13.7**	9.69
TE_Test Iscador M 5 mg_0.1 µg/mL	0.083094595	83.9	**93.0**	**12.9**	9.14
	Peak Area Ratio	% metabolite vs NC			
E_Negative Control 1-1_no inhibitor	0.089878988	90.7	**93.8**	**4.32**	3.06
E_Negative Control 1-2_no inhibitor	-	-	-	-	-
E_Negative Control 1-3_no inhibitor	0.108213975	109.3	**106.2**	**4.32**	2.50
ative Control 2_no inhibitors/no micr	0.00	0.00	**0.0**	**0.00**	0.00
Control 3_no inhibitors/no microsom	0.00	0.00	**0.0**	**0.00**	0.00
	Peak Area Ratio	% metabolite vs NC			
Average Negative controls	0.099046481	100	**100.0**	**0.00**	0.00

Data Fig S5_CYP3A4-5 inhibition assays by Ketoconaole_Endoxifen_Metabolite_Mean of 3 assays_SD_SE

Assay I			Assay II		
Endoxifen (metabolite)			Endoxifen (metabolite)		
	Peak Area Ratio	% metabolite vs NC		Peak Area Ratio	% metabolite vs NC
TE_Positive Control_KETOCONAZOLE 100 uM	1.54127E-06	3.73	TE_Positive Control_KETOCONAZOLE 100 uM	0.00	0.00
TE_Positive Control_KETOCONAZOLE 10 uM	7.62948E-06	18.5	TE_Positive Control_KETOCONAZOLE 10 uM	4.26237E-06	10.67
TE_Positive Control_KETOCONAZOLE 5 uM	1.02207E-05	24.7	TE_Positive Control_KETOCONAZOLE 5 uM	-	-
TE_Positive Control_KETOCONAZOLE 2 uM	1.1345E-05	27.5	TE_Positive Control_KETOCONAZOLE 2 uM	1.52932E-05	38.30
TE_Positive Control_KETOCONAZOLE 1 uM	1.62545E-05	39.4	TE_Positive Control_KETOCONAZOLE 1 uM	2.38592E-05	59.7
TE_Positive Control_KETOCONAZOLE 0.1 uM	4.00021E-05	96.9	TE_Positive Control_KETOCONAZOLE 0.1 uM	-	-

Assay III			Mean	SD	SE
Endoxifen (metabolite)					
	Peak Area Rati	% metabolite vs NC			
TE_Positive Control_KETOCONAZOLE 100 uM	0.00	0.00	1.24	2.15	1.244
TE_Positive Control_KETOCONAZOLE 10 uM	5.39611E-06	12.36	13.8	4.10	2.370
TE_Positive Control_KETOCONAZOLE 5 uM	9.03534E-06	20.69	22.7	2.87	2.028
TE_Positive Control_KETOCONAZOLE 2 uM	1.68183E-05	38.5	34.8	6.31	3.646
TE_Positive Control_KETOCONAZOLE 1 uM	2.18172E-05	50.0	49.7	10.20	5.888
TE_Positive Control_KETOCONAZOLE 0.1 uM	-	-	96.9	-	-

Data Fig S6_CYP3A4-5 inhibition assays by Ketoconazole_6BetaHT_Metabolite_Mean of 3 assays_SD_SE

Assay I			Assay II		
Endoxifen (metabolite)			Endoxifen (metabolite)		
	Peak Area Ratio	% metabolite vs NC		Peak Area Ratio	% metabolite vs NC
TE_Positive Control_KETOCONAZOLE 100 uM	0.000544197	4.17	TE_Positive Control_KETOCONAZOLE 100 uM	0.00	0.00
TE_Positive Control_KETOCONAZOLE 10 uM	0.000649502	4.97	TE_Positive Control_KETOCONAZOLE 10 uM	0.00	0.00
TE_Positive Control_KETOCONAZOLE 5 uM	0.001170492	8.96	TE_Positive Control_KETOCONAZOLE 5 uM	0.000928438	4.77
TE_Positive Control_KETOCONAZOLE 2 uM	0.001259259	9.64	TE_Positive Control_KETOCONAZOLE 2 uM	0.001192394	6.13
TE_Positive Control_KETOCONAZOLE 1 uM	0.002362481	18.08	TE_Positive Control_KETOCONAZOLE 1 uM	0.002148023	11.04
TE_Positive Control_KETOCONAZOLE 0.1 uM	0.005440135	41.64	TE_Positive Control_KETOCONAZOLE 0.1 uM	-	-

Assay III			Mean	SD	SE
Endoxifen (metabolite)					
	Peak Area Ratio	% metabolite vs NC			
TE_Positive Control_KETOCONAZOLE 100 uM	0.003171883	3.20	2.46	2.18	1.259
TE_Positive Control_KETOCONAZOLE 10 uM	0.004942901	4.99	3.32	2.88	1.660
TE_Positive Control_KETOCONAZOLE 5 uM	0.002599058	2.62	5.45	3.22	1.860
TE_Positive Control_KETOCONAZOLE 2 uM	-	-	7.88	2.48	1.756
TE_Positive Control_KETOCONAZOLE 1 uM	0.015401216	15.55	14.89	3.57	2.061
TE_Positive Control_KETOCONAZOLE 0.1 uM	0.047996546	48.46	45.05	4.82	3.411
