# Supplementary figures and images for: Absence of herb-drug interactions of mistletoe with the tamoxifen metabolite (E/Z)-endoxifen and cytochrome P450 3A4/5 and 2D6 in vitro
Source: BMC Complement Altern Med. 2019 Jan 18;19:23. doi: 10.1186/s12906-019-2439-2 (PMC6339413; doi:10.1186/s12906-019-2439-2)

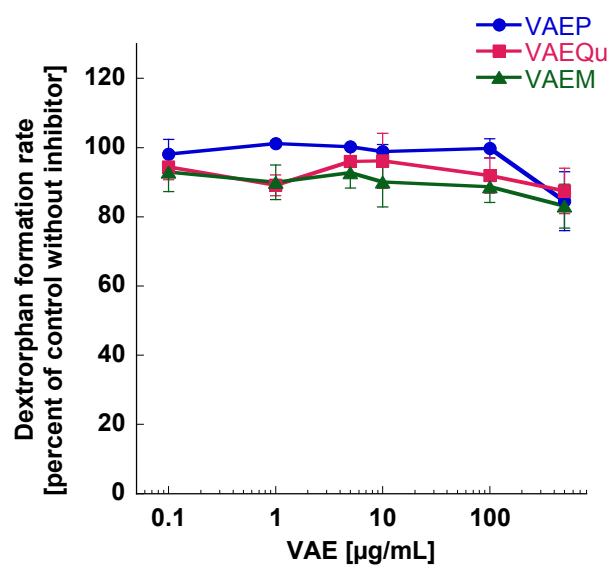

Supplement: Supplementary file 4 — Figure S1. Dextromethorphan in vitro inhibition profiles of CYP2D6 by mistletoe preparations (VAEP, VAEQu, and VAEM). Results are presented as mean rate values ± SE (from three independent experiments) of dextrorphan formation in vitro expressed as a percent of the control without inhibitor. Significance values are given relative to the negative controls without inhibitors (*p<0.05, **p < 0.01, ***p < 0.001). (PDF 36 kb) [file 12906_2019_2439_MOESM4_ESM.pdf]

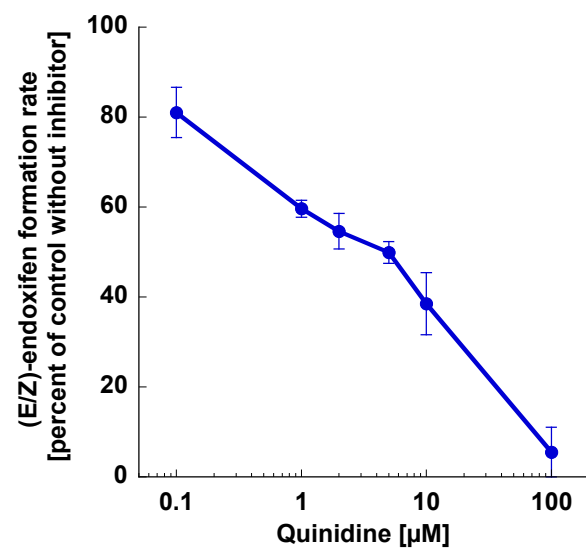

Supplement: Supplementary file 5 — Figure S2. Tamoxifen in vitro inhibition profiles of CYP2D6 by reference inhibitor: Quinidine (0.1-1-2-5-10-100 μM). Results are presented as mean rate values ± SE (from three independent experiments) of (E/Z)-endoxifen formation in vitro expressed as a percent of the control without inhibitor. (PDF 27 kb) [file 12906_2019_2439_MOESM5_ESM.pdf]

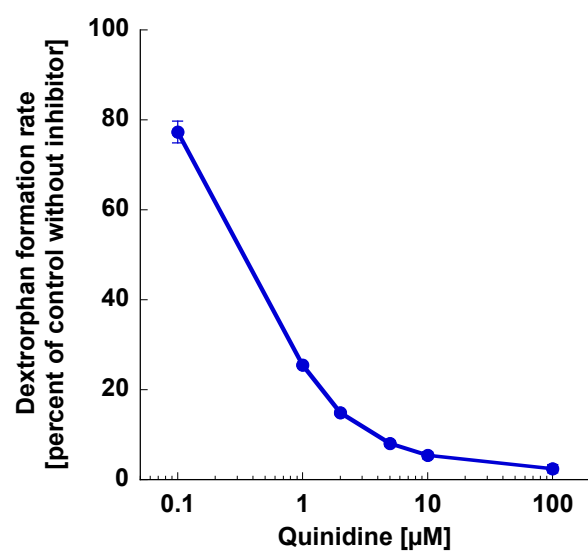

Supplement: Supplementary file 6 — Figure S3. Dextromethorphan in vitro inhibition profiles of CYP2D6 by reference inhibitor: Quinidine (0.1-1-2-5-10-100 μM). Results are presented as mean rate values ± SE (from three independent experiments) of dextrorphan formation in vitro expressed as a percent of the control without inhibitor. (PDF 26 kb) [file 12906_2019_2439_MOESM6_ESM.pdf]

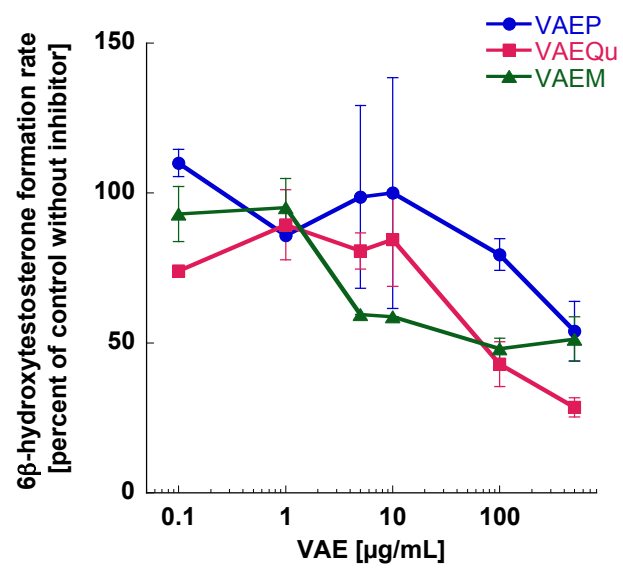

Supplement: Supplementary file 7 — Figure S4. Testosterone in vitro inhibition profiles of CYP3A4/5 (VAEP, VAEQu, VAEM). Results are presented as mean rate values ± SE (from three independent experiments) of 6β-hydroxytestosterone formation in vitro expressed as a percent of the control without inhibitor. (PDF 40 kb) [file 12906_2019_2439_MOESM7_ESM.pdf]

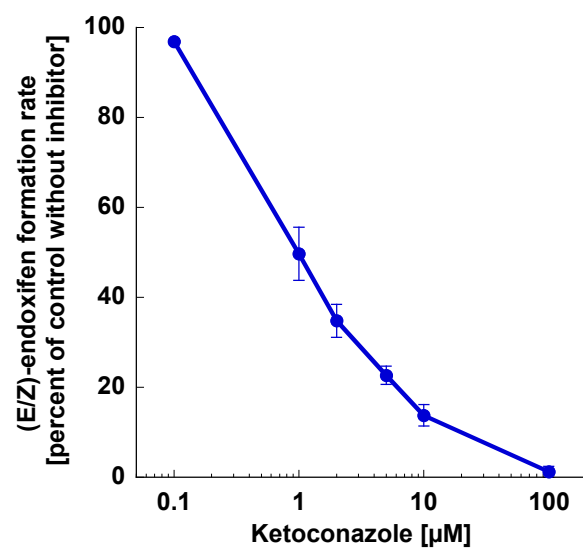

Supplement: Supplementary file 8 — Figure S5. Tamoxifen in vitro inhibition profile of CYP3A4/5 by reference inhibitor: Ketoconazole (0.1-1-2-5-10-100 μM). Results are presented as mean rate values ± SE (from three independent experiments) of (E/Z)-endoxifen formation in vitro expressed as a percent of the control without inhibitor. (PDF 27 kb) [file 12906_2019_2439_MOESM8_ESM.pdf]

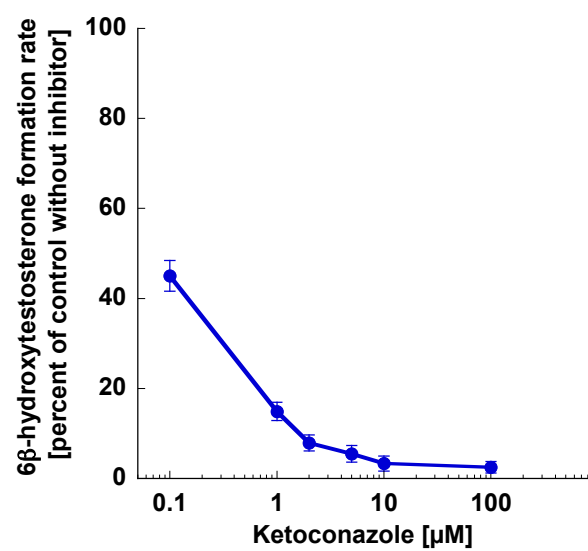

Supplement: Supplementary file 9 — Figure S6. Testosterone in vitro inhibition profile of CYP3A4/5 by reference inhibitor: Ketoconazole (0.1-1-2-5-10-100 μM). Results are presented as mean rate values ± SE (from three independent experiments) of 6β-hydroxytestosterone formation in vitro expressed as a percent of the control without inhibitor. (PDF 31 kb) [file 12906_2019_2439_MOESM9_ESM.pdf]

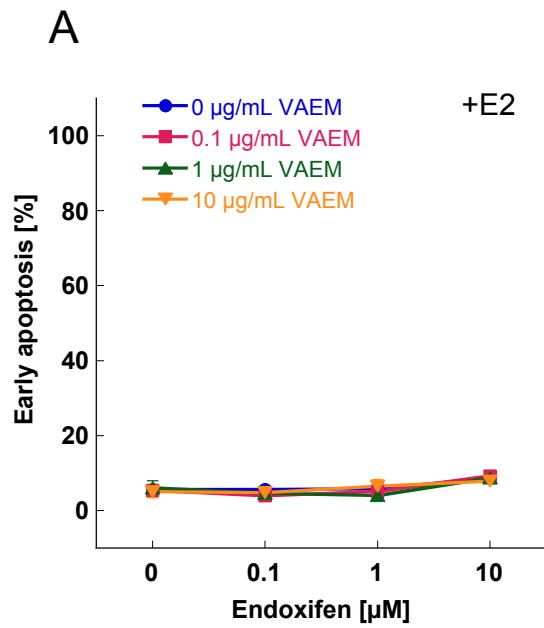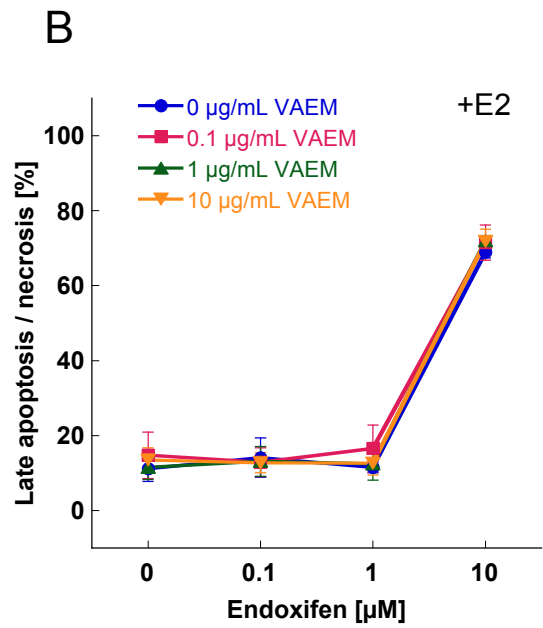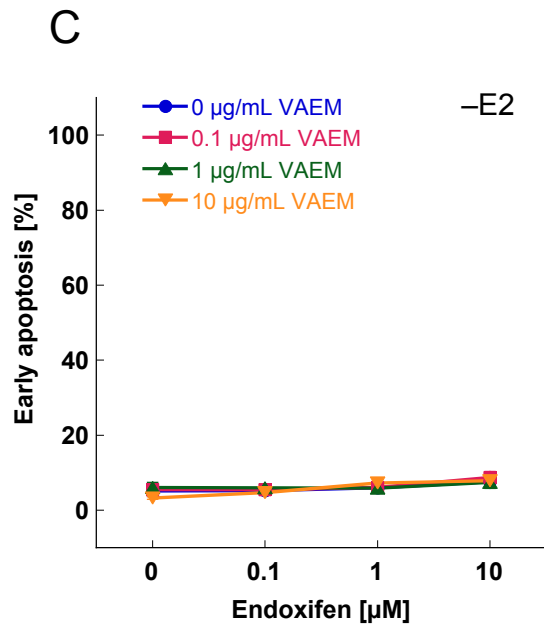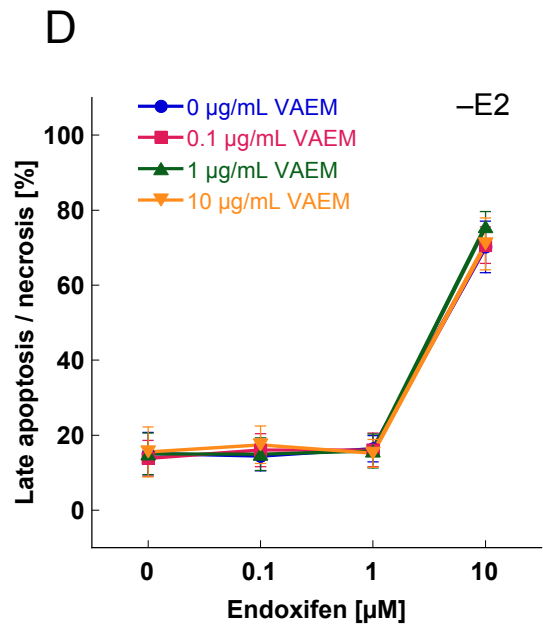

Supplement: Supplementary file 10 — Figure S7. Apoptosis induction (%) in MCF-7 cells after 3d treatment with endoxifen in combination with VAEM. Mean values (±SE) of (A) early apoptosis in the presence of 0.5 μM β-estradiol (E2) (B) late apoptosis/necrosis in the presence of 0.5 μM β-estradiol, (C) early apoptosis in the absence of 0.5 μM β-estradiol and (D) late apoptosis/necrosis in the absence of 0.5 μM β-estradiol are presented (*p < 0.05, **p < 0.01, ***p < 0.001). (PDF 53 kb) [file 12906_2019_2439_MOESM10_ESM.pdf]
